# Retail-Level Microbiomes of Organic and Conventional Fresh Produce: A Multi-Kingdom Analysis of Amoeba-Associated Bacterial Viability

**DOI:** 10.3390/foods15122230

**Published:** 2026-06-20

**Authors:** Lara Soler, Laura Moreno-Mesonero, Jorge García-Hernández, Miguel García-Ferrús, Andrés Zornoza, Yolanda Moreno

**Affiliations:** 1 Instituto de Ingeniería del Agua y Medio Ambiente (IIAMA), Universitat Politècnica de València, Camino de Vera s/n, 46022 València, Spain; lasogar@etsiamn.upv.es (L.S.);; 2 Centro Avanzado de Microbiología Aplicada (CAMA), Universitat Politècnica de València, Camino de Vera s/n, 46022 València, Spain; jorgarhe@btc.upv.es (J.G.-H.);

**Keywords:** fresh produce microbiome, foodborne pathogens, food safety, consumer exposure, retail-level risk

## Abstract

The increasing consumption of fresh organic produce has given rise to concerns regarding the microbiological safety of minimally processed foods. Organic cultivation may be associated with increased exposure to environmental microorganisms due to soil-based inputs and reduced chemical interventions, including both beneficial taxa and potential foodborne pathogens. Fresh produce is known to harbour complex microbial ecosystems, which are shaped by farming practices, plant physiology, handling, packaging and storage, particularly in raw-consumed products such as leafy greens and strawberries. In this study, bacterial (16S rRNA) and eukaryotic (18S rRNA) communities were characterized by amplicon sequencing. In parallel, an amoeba-associated bacterial microbiome was analyzed and DVC-FISH was used to assess the viability and metabolic activity of pathogenic bacteria internalized within free-living amoebae (FLA). No significant differences in alpha or beta diversity were observed between organic and conventional products, suggesting microbiome convergence at the retail stage driven by post-harvest handling and processing. Potentially pathogenic genera, including *Pseudomonas*, *Stenotrophomonas*, and *Acinetobacter* (bacterial), as well as *Tilletiopsis*, *Candida*, and *Naegleria* (eukaryotic), were identified in both organic and non-organic microbiomes. The viability of FLA-internalized *Pseudomonas* spp. was confirmed by DVC-FISH, demonstrating that FLA act as reservoirs, enhancing pathogen persistence in fresh produce. This integrated assessment of organic and conventional fruits and vegetables at the retail stage highlights the importance of post-harvest handling and retail conditions in shaping microbiological safety. The integration of microbiome profiling with targeted viability analyses demonstrates that downstream stages are critical control points for food safety and consumer exposure, beyond the influence of the production system alone.

## 1. Introduction

In recent years, there has been a global increase in the preference for organic food products among consumers [1] and the scale of organic production has exhibited remarkable growth. The practice of organic farming in 2024 was observed in 188 countries across the globe, encompassing a total area of 96 million hectares of agricultural land, which represents a growth of 5.3 times the recorded area in 1999 [2].

Organic farming is based on the concept of ecological production management. This approach emphasizes the use of crop rotation, natural pest management, crop and livestock diversification, and soil improvement through plant and animal fertilizers [3,4,5,6].

According to United Nations projections, the global population is expected to reach 9.7 billion by 2050. This demographic shift underscores the growing significance of organic production, as the increase in both population size and life expectancy are accompanied by an increased demand for not only traditional food products but also high-quality alternatives [7]. Furthermore, an increase in the consumption of organic products is expected in coming years, as consumer trends have shifted, showing a growing demand for products that are fully prepared outside the home and minimally processed. This phenomenon has resulted in the development of a category of products, designated as Range IV, which includes ready-to-eat salads (RTESs) [8,9,10]. It is estimated that the organic packaged salad segment will experience a compound annual growth rate (CAGR) of 8.3% from 2024 to 2030 [11].

Consumers tend to perceive organic food products as healthier, tastier, and of higher quality than conventional foods [12,13]. However, both fresh fruits and vegetables produced through conventional and organic farming practices can become contaminated with pathogens at various points during the production process [14]. The prevalence of pathogens in fresh vegetables represents a significant public health concern, particularly in products that are consumed raw [15]. Recent outbreaks of foodborne illness in Europe and the USA have demonstrated a correlation between certain pathogens and specific leafy green vegetables, including lettuce and spinach in ready-to-eat salads [16]. The utilization of untreated irrigation water and unsuitable organic fertilizers represents a considerable risk factor for the microbiological contamination of fresh leafy green vegetables [17,18]. Manure is frequently used as a fertilizer in organic vegetable production, which, if not properly processed, can result in the contamination of crops due to the potential presence of pathogenic bacteria, including *Salmonella* spp., *Listeria monocytogenes*, *Escherichia coli* O157:H7, and other verotoxin-producing bacteria [19].

Evidence indicates that different agricultural systems can modify the bacterial communities present in crops [20,21]. However, the microbiome associated with fresh fruits and vegetables is not solely determined by cultivation practices but is progressively shaped by post-harvest processes occurring along the supply chain. During the supply chain, factors such as transportation, storage conditions, handling, and cross-contamination at the point of sale may influence the composition of the microbiome [22,23]. Despite these findings, most previous studies have focused on specific pathogens, indicators or resistance traits, providing limited insight into the overall microbial ecology of fresh produce as purchased by consumers. Such approaches may overlook microbiome-level changes occurring after harvest, including community convergence and ecological interactions that promote microbial persistence.

There is also limited information regarding the role of free-living amoebae (FLA) as carriers or promoters of bacterial pathogen associated risk in fresh produce. FLA are widely recognized as environmental reservoirs for a broad range of pathogenic bacteria, which can survive and replicate intracellularly [24], enhancing bacterial resistance to environmental stresses, disinfectants, and host immune defences, and thereby increasing their persistence and virulence [25]. The presence of free-living amoebae (FLA) has been previously identified in raw vegetables [26,27]. Consequently, the characterization of FLA-associated microbiomes is critical to better understand their role in environmental persistence, transmission, and potential public health risk of pathogenic bacteria in fresh produce.

Characterizing the total microbiome of fresh fruits and vegetables at the retail level is essential to understand how agricultural practices, post-harvest handling and microbial ecology jointly shape realistic consumer exposure and provide a more integrative and relevant framework for food safety assessment than evaluations restricted to the production stage. Thus, the aim of the present study was to evaluate the differences in microbial composition between fresh organic and conventional products. A comparative analysis of the bacterial and eukaryotic, as well as amoeba-associated microbiome, was conducted through amplicon sequencing. Furthermore, the presence and viability of potentially pathogenic bacteria within the microbiome of isolated FLA was assessed by applying the molecular Direct Viable Count–Fluorescence in situ Hybridization (DVC-FISH) technique.

## 2. Materials and Methods

### 2.1. Samples

A total of 154 samples, including cabbage (C), spinach (Sp), strawberry (St), and lettuce (L), were collected between November 2020 and April 2023. The samples were purchased from 45 local retailers, including stores specialized in the sale of organic products in the city of Valencia (Table S1). The samples included both organic (*n* = 114) and conventional products (*n* = 40). In all cases, fresh, unpackaged products were obtained. Samples were transported in sterile sealed bags at 4 °C and processed within 24 h of collection. Before processing leafy green vegetables, the outer leaves were discarded for analysis [28].

Three distinct analyses were conducted on each sample: (i) characterization of the bacterial microbiome by 16S rRNA gene sequencing; (ii) characterization of the eukaryotic microbiome by 18S rRNA gene sequencing; and (iii) analysis of the bacterial microbiome associated with FLA (bacterial communities that are internalized within amoebae cells).

Furthermore, FLA were isolated from the 154 samples and analyzed by DVC-FISH, to study the presence and viability of potentially pathogenic bacteria within the isolated FLA (Table S1).

### 2.2. Bacterial Microbiome

Each sample (25 g) was mixed with 225 mL of buffered peptone water in a sterile blender bag (Bag Page, Intescience, Saint-Nom-la-Bretèche, France) and homogenized for one minute at medium speed using the Mayo Homogenius HG 400 homogenizer. The strawberry samples were only shaken horizontally for five minutes to ensure proper elution of microorganisms. This modification was necessary because stomacher processing of strawberries produced a thick paste that interfered with subsequent DNA extraction. The resulting homogenates were subjected to centrifugation for a period of 10 min at 8000 rpm; the pellets were resuspended in 1 mL of phosphate-buffered saline (PBS) solution and stored at −80 °C until DNA extraction. DNA was extracted using the High Pure PCR Template Preparation Kit (Roche, Grenzach-Wyhlen, Germany), in accordance with the manufacturer’s instructions. DNA was stored at −20 °C until 16S rRNA library preparation and amplicon sequencing were performed.

### 2.3. Eukaryotic Microbiome

A recommended amount for protozoa detection of 100 g per sample was combined with 200 mL of a detergent solution (1× PBS, 0.1% Tween 80, 0.1% sodium dodecyl sulfate (SDS), and 0.05% antifoam emulsion), in accordance with the methodology described by Moreno-Mesonero et al. (2023) [27]. The mixture was subjected to homogenization as described above. The same quantity of detergent solution was employed for the strawberry samples, which were horizontally shaken for 10 min. The homogenates were then subjected to centrifugation at 2000 rpm for 15 min, after which they were resuspended in 1 mL of PBS and stored at −80 °C until DNA extraction. DNA extraction was conducted using the FastDNA™ Spin Kit for Soil (MP Biomedicals, Irvine, CA, USA), along with Lysing Matrix E tube and FastPrep-24^®^ instrument (MP Biomedicals, Irvine, CA, USA) for the homogenization step. DNA was stored at −20 °C until 18S rRNA library preparation and amplicon sequencing were performed.

### 2.4. Bacterial Microbiome Associated with Isolated Free-Living Amoeba (FLA)

One hundred grams of leafy green vegetable samples were placed in a sterile blender bag with 250 mL of Page’s amoeba saline (PAS) buffer. The mixture was then homogenized for one minute at medium speed. Similarly, 100 g of strawberries were combined with 250 mL of PAS buffer and gently homogenized by shaking for 10 min. The homogenates were filtered through sterile nitrocellulose membranes with a pore size of 1.2 µm and placed upside-down into non-nutrient agar (NNA) plates. The plates were incubated at 28 °C for 24 h, then the filter was removed, and the plates were maintained at this temperature until FLA growth was observed, or for a maximum of 30 days. FLA mixed cultures were recovered using a sterile cell scraper. The solution containing all the FLA present on the plates was then subjected to centrifugation at 500 g for 3 min, and the pellet was resuspended in 500 μL of PBS. Subsequently, a sodium hypochlorite solution was added to achieve a final concentration of 100 ppm, with the objective of inactivating any bacteria outside FLA, maintaining the solution under aerobic conditions and in the dark for one hour. After that, it was washed with PBS and centrifuged at 500 g for three minutes, to eliminate residual sodium hypochlorite. Finally, the pellet was resuspended in 1 mL of PBS. A portion 500 μL was treated with Propidium Monoazide (PMA) to prevent DNA of non-internalized bacteria from amplification as previously described [29] and used for DNA isolation, while another 500 mL portion was processed for DVC-FISH analysis. DNA was extracted using the GeneJET™ Genomic DNA Purification Kit (Thermo Scientific, Bremen, Germany), and DNA was stored at −20 °C until 16S rRNA amplicon sequencing was performed. A specific qPCR assay was used to identify *Acanthamoeba* sp. among the FLA cultures according to Moreno-Mesonero et al. (2023) [27]. Briefly, detection of *Acanthamoeba* spp. targeted the 18S rRNA gene using the primers Acant900-F (5′-CCCAGATCGTTTACCGTGAA-3′) and Acant1100-R (5′-TAAATATTAATGCCCCCAACTATCC-3′), and the probe Acant1000-P (5′-6-FAM-CTGCCACCGAATACATTAGCATGG-BHQ1-3′), yielding a 180 bp amplicon. Thermal cycling conditions consisted of an initial denaturation at 95 °C for 10 min, followed by 40 cycles of 95 °C for 10 s, 63 °C for 8 s, and 72 °C for 7 s. Reaction and data analysis were performed using a LightCycler 2.0 system (Roche, Barcelona, Spain) with the LightCycler TaqMan Master kit (Roche).

### 2.5. Amplicon Sequencing and Data Analysis

Amplicon sequencing was conducted by FISABIO Sequencing Service (Valencia, Spain) using paired-end 2 × 300 bp sequencing on the Illumina MiSeq platform.

To analyze the total bacterial communities and those associated with FLA, amplicon libraries were created using primers and conditions specified in the 16S Metagenomic Sequencing Library Preparation Guide. These targeted the V3-V4 region of the 16S rRNA gene, producing a single 460 bp amplicon (Part #15044223 Rev. B). To sequence the eukaryotic microbiome, the V4 hypervariable region of the 18S rRNA gene was targeted with EUKAF and EUKAR primers, as previously described by Moreno et al. (2018) [30]. Quality assessment was performed by the Sequencing Service using the fastp programme, applying the following parameters: minimum reads length: 50; minimum trimming quality mean: 20; over a window of: 10 bp. When a sequence does not fit the minimum standards of length and/or quality, it is removed from the dataset. Negative controls were routinely included in all sequencing runs to monitor for contamination, as part of the Sequencing Service provider’s quality control procedures.

The raw sequencing data were processed using the QIIME2 v2024.10 software (https://qiime2.org/ (accessed on 7 April 2026), [31]). The sequences were initially imported into the software, after which the forward and reverse reads were merged. Quality filtering and chimera checks were applied, with the objective of removing any sequences derived from chloroplasts, mitochondria and higher plants and the remaining sequences were denoised and grouped into amplicon sequence variants (ASVs) using the DADA2 algorithm [32]. Sequences below 200 bp or with a Phred quality score below Q30 were excluded. The taxonomic classification of ASVs was conducted through the Naive Bayes classifier in QIIME2, utilizing SILVA 138 reference sequences [33].

### 2.6. Statistical Analysis

Prior to diversity analyses, samples were rarefied to account for differences in sequencing depth. Alpha diversity was assessed using two metrics: observed features, as an estimate of community richness, and Faith’s phylogenetic diversity index [27], as a measure incorporating phylogenetic relationships among taxa. Differences in alpha diversity among sample types were evaluated using the Kruskal–Wallis test [34].

To explore beta diversity patterns, relative abundance data were first square-root transformed to downweight the contributions of quantitatively dominant taxa. A Bray–Curtis resemblance matrix was then generated [35]. Non-metric multidimensional scaling (nMDS) was used to visualize distribution patterns of bacterial and eukaryotic communities according to sample type.

To formally test the null hypothesis of no differences among these a priori defined sample types, a one-way analysis of similarities (ANOSIM) was applied directly to the resemblance matrix. This avoids the circularity of testing the visual groups formed in the nMDS space. ANOSIM was performed using 999 permutations and provided the global R statistic as an absolute measure of effect size, pairwise R values, and their associated significance levels.

According to Clarke et al. (2014) [36], the ANOSIM R statistic measures the degree of separation among groups, with R values close to 1 indicating complete separation and values close to 0 indicating no separation. In this study, R values > 0.75 were interpreted as indicating good separation among groups, R values > 0.50 as indicating separated groups, and R values < 0.25 as indicating groups that were barely separated or highly overlapping.

As an additional complementary analysis to ANOSIM, permutational multivariate analysis of variance (PERMANOVA) was used to test the null hypothesis of no significant differences in the spatial location, or centroids, of the communities among groups [37]. PERMANOVA was also performed using 999 permutations. All multivariate statistical routines were carried out using PRIMER v7 [38] with the PERMANOVA+ add-on [37].

*p*-values obtained from ANOSIM were not adjusted for multiple comparisons. Therefore, matrix-specific pairwise comparisons were interpreted cautiously and considered exploratory. In all cases, statistical significance was not evaluated solely on the basis of *p*-values, but was interpreted together with the ANOSIM R statistic, which was used as an effect size measure of the magnitude of group separation.

For genus-level comparisons showing statistically significant ANOSIM results together with moderate or higher R values, similarity percentage analysis (SIMPER) was used to identify the genera contributing most to the observed Bray–Curtis dissimilarity between organic and non-organic samples. SIMPER was applied only to those comparisons showing a meaningful degree of group separation, whereas comparisons with low ANOSIM R values were not subjected to SIMPER because they indicated substantial overlap between groups.

A significance level of *p* < 0.05 was used for all statistical analyses.

### 2.7. Direct Viable Count Fluorescent In Situ Hybridization (DVC-FISH) of Pathogenic Bacteria Associated with Free-Living Amoebae (FLA)

All samples were analyzed to identify the presence and viability of *L. monocytogenes*, *Helicobacter* spp., *Salmonella* spp. and *Pseudomonas* spp. in the FLA by DVC-FISH.

Briefly, each sample resuspended in PBS (Section 2.4) was incubated in different media depending on the type of microorganism tested. For the detection of viable *L. monocytogenes*, the sample solution was added to 5 mL of DVC-Lm medium (37 g/L brain hearth infusion (BHI) broth with 2.5 mg/mL yeast extract and 2 mg/mL ciprofloxacin) and incubated for 7 h at 37 °C [39]. For *Helicobacter* spp., each sample was added to 5 mL of DVC-H medium (28 g/L BBL™ Brucella broth supplemented with 5% fetal bovine serum (FBS) and 0.5 mg/L novobiocin) and incubated for 24 h at 37 °C under microaerophilic conditions (5% O_2_, 10% CO_2_ and 85% N_2_) [40]. For *Pseudomonas* spp. and *Salmonella* spp., each sample was added to 5 mL of DVC-Ps/S medium (13 g/L lactose broth with 2.5 g/L yeast extract and 40 μg/mL nalidixic acid) and incubated for 18 h at 37 °C [41]. After incubation, samples were centrifuged at 7000 rpm for 3 min, and the supernatant was removed and washed with PBS.

For fixing Gram-negative bacteria (*Helicobacter* spp., *Pseudomonas* spp. and *Salmonella* spp.), the pellet was resuspended in a 1:3 ratio of PBS:PFA (paraformaldehyde) and incubated at 4 °C for 3 h. Samples were then centrifuged at 7000 rpm for 3 min, the pellet was resuspended in 1:1 PBS:100% ethanol and stored at −20 °C until hybridization. For Gram-positive bacteria (*L. monocytogenes*), the pellet was directly resuspended in a 1:1 PBS:100% ethanol and stored at −20 °C until hybridization was performed.

An aliquot of 10 µL of fixed samples was placed in gelatin-coated slides. For Gram-positive bacteria (*L. monocytogenes*), 10 μL of 50 mg/mL lysozyme was added and incubated for 10 min at 4 °C, after which the slides were washed with distilled water. All the slides were then immersed in volumes of 50, 80 and 100% ethanol for 3 min each. Once the slides were dried, 10 μL of hybridization buffer (0.9 M NaCl, 20 mM HCl-Tris, 0.01% SDS and 30% formamide, pH 7.5) containing 50 ng of each of the probes labelled with the corresponding fluorochrome (Table S2, [42,43,44,45,46]) was added to each well. The reaction was carried out in the dark at 46 °C for 1.5 h according to Moreno et al. (2001) [47]. The slides were then washed with 50 mL of washing solution (0.10 M NaCl, 0.02 M HCl-Tris, 0.01% SDS and 0.005 M EDTA) and tempered at 48 °C for 15 min in the dark. Following this, the samples were washed with distilled water and air-dried in the dark. The slides were mounted with FluoroGuard Antifade Reagent (Bio-Rad, Spain) between the coverslip and visualized using an Olympus BX 50 fluorescence microscope with U-MWB, U-MWIB and U-MWIG filters. A pure fixed cell culture of DVC-incubated *L. monocytogenes* (CECT 4032), *Helicobacter pylori* (NCTC 11637), *Salmonella enterica* subsp. *enterica* (CECT 443). and *P. aeruginosa* (CECT 108) were used as positive controls for the DVC-FISH reaction. To consider a positive result, the size of target bacteria should be similar to that of DVC-incubated positive controls. Moreover, a negative control with no sample was included in all assays.

## 3. Results and Discussion

The worldwide increase in demand for organic foods is mainly due to growing health concerns and the perception that natural products are safer options [48]. However, the microbiological safety and microbial composition of organic produce remain under-explored. Although some studies [49] provided pioneering characterization of bacterial communities associated with organic and conventional produce at the retail level, our study extends this work by integrating multi-kingdom microbiome profiling and the assessment of pathogen viability and persistence within free-living amoebae. This approach offers a more comprehensive and risk-oriented perspective of fresh produce microbiology.

In this work, we have analyzed the microbial communities present in a selection of organic and conventional vegetable food samples, including cabbage, spinach, strawberries and lettuce, to identify differences that may be attributed to cultivation practices. These products were selected because they have been identified as having some of the highest levels of pesticide residue among fruits and vegetables [50], and because they are usually consumed raw. Therefore, if their microbiological quality is not compromised, consuming these organic products may be a healthier option. A larger number of organic samples than conventional ones were included owing to their lower market availability and the limited number of studies addressing these products. Nevertheless, the overall sample size was sufficient to support statistically significant comparative analyses.

### 3.1. Bacterial Microbiome

Following the sequencing process, a total of 14,025,326 raw reads were generated. After the implementation of quality filtering, the removal of chimeric sequences, and the exclusion of chloroplast and mitochondrial sequences, a total of 4,595,968 reads were retained. The reads were then rarefied and grouped into 1216 amplicon sequence variants (ASVs) (Table S3). It should be noted that not all samples were included in the analysis, due to the low number of sequencing reads after amplicon sequencing (Table S1).

**Figure 1:**
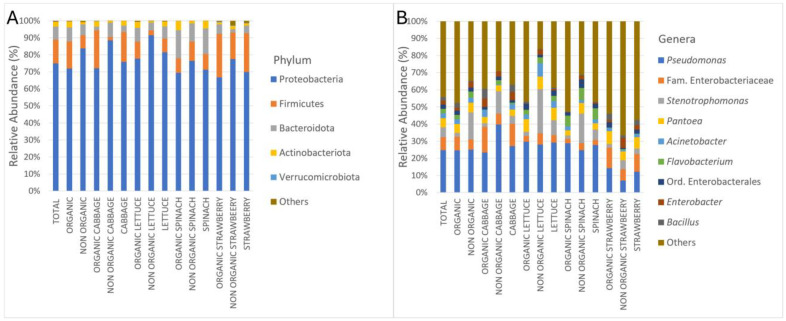
Relative abundance (%) of bacterial communities in organic and non-organic products at the phylum (**A**) and genus (**B**) levels.

At the phylum level, the most prevalent phyla were Proteobacteria (75.99%), Firmicutes (13.90%), Bacteroidota (7.66%) and Actinobacteriota (2.87%) (Figure 1A). At the genus level, the predominant taxa included *Pseudomonas* (24.70%), some genera of the Enterobacteriaceae family (7.54%), *Stenotrophomonas* (5.77%), and *Pantoea* (5.36%) (Figure 1B). Some studies on conventional fruits and vegetables previously found that Proteobacteria was the most dominant phyla and identified *Pseudomonas* and genera of Enterobacteriaceae family as being particularly abundant [51,52,53]. In this study, the relative abundance of the genus *Pseudomonas* was similar between organic (24.66%) and non-organic (25.0%) samples.

Alpha diversity analysis is a commonly employed method for characterizing two pivotal aspects of microbial communities: community richness, which refers to the number of taxonomic groups present (i.e., the number of distinct species or genera), and evenness, which reflects the distribution of these group abundances within the community. Richness is indicative of the overall biodiversity, whereas evenness demonstrates whether some species are dominant or whether all species are similarly represented in terms of number of species or genera [54,55]. In the current study, alpha diversity results indicated that organic product samples exhibited a greater number of observed features in comparison to non-organic products (Figure S1A). This observation is further supported by the findings of the Faith’s phylogenetic diversity index, which revealed statistically significant differences between the two types of samples (Kruskal–Wallis test, *p* = 0.0068, Table S4, Figure S1B).

Beta diversity analysis was performed to evaluate differences in bacterial community composition among sample types using ANOSIM. At the global level, organic and non-organic samples showed statistically significant differences but weak separation at the phylum level (R = 0.148, *p* = 0.002; Table S6), indicating substantial overlap between both production systems. At the genus level, the separation was slightly higher but remained moderate to weak (R = 0.288, *p* = 0.001; Table S8).

When samples were analyzed by matrix type, ANOSIM revealed significant but generally weak separation at the phylum level, with R values below 0.25 for lettuce, spinach, and strawberry, and a higher but still moderate value for cabbage (R = 0.351, *p* = 0.001; Table S7). At the genus level, clearer matrix-specific patterns were observed. Organic and non-organic cabbage (R = 0.551, *p* = 0.001) and strawberry samples (R = 0.570, *p* = 0.001) showed the strongest separation, whereas lettuce showed moderate separation (R = 0.364, *p* = 0.001) and spinach showed weak separation (R = 0.226, *p* = 0.001; Table S9). These results indicate that production system-related differences in the bacterial microbiome were not strongly expressed at the global level, but became more evident in specific produce types, particularly at the genus level.

Accordingly, SIMPER analysis was applied to the genus-level comparisons showing the most meaningful ANOSIM separation in the bacterial microbiome, namely cabbage, lettuce, and strawberry samples. The genera contributing most to the dissimilarity between organic and non-organic products are shown in Table S10. Briefly, the main contributors included *Pseudomonas*, *Stenotrophomonas*, *Enterobacter*, *Bacillus*, *Bradyrhizobium*, *Mesorhizobium*, *Pantoea*, *Serratia*, *Lactococcus*, *Acinetobacter*, and *Exiguobacterium*, although their relative contribution and direction of change varied depending on the produce type.

**Figure 2:**
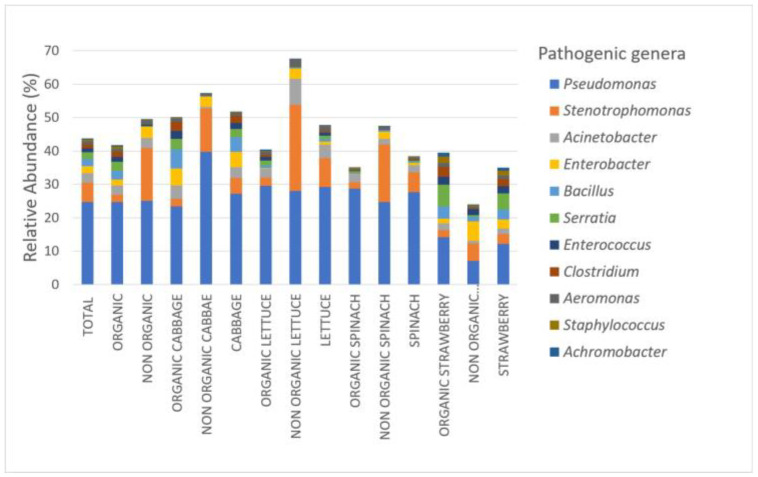
Relative abundance (%) of clinically relevant bacterial genera in organic and non-organic products.

The presence of certain potentially pathogenic bacteria was determined (Figure 2). The most prevalent genera were *Pseudomonas* (24.70%), *Stenotrophomonas* (5.77%), *Acinetobacter* (2.83%) and *Enterobacter* (2.21%), some species of which are known to cause human opportunistic diseases such as pneumonia (*P. aeruginosa*), nosocomial infections such as pneumonia and septicemia (*Stenotrophomonas maltophilia*), urinary tract infections and bacteremia (*Acinetobacter baumannii*), and septicemia and meningitis (*Enterobacter cloacae*) [56,57,58,59]. This information is relevant given the documentation of outbreaks traced to the consumption of fresh lettuce contaminated with *Pseudomonas* spp. and *Enterobacteriaceae* [53,60].

The prevalence of *Stenotrophomonas* was found to be 15.93% in non-organic produce compared to 2.15% in organic produce (Figure 2). The significantly higher prevalence of *Stenotrophomonas* in non-organic produce may be linked to conventional agricultural practices, which is associated with lower microbial diversity in soil. By contrast, organic farming promotes more balanced microbial ecosystems and enforces stricter controls on inputs, which may prevent opportunistic bacteria such as *Stenotrophomonas* from becoming established [61,62]. Conversely, the relative abundance of *Bacillus*, *Enterococcus*, and *Clostridium* was found to be higher in organic products (<2.6%) compared to conventional ones (<0.5%). The presence of *Bacillus* in organic products is expected, as it is used as a biopesticide to control various phytopathogens [63,64]. The use of animal manure in organic farming has been demonstrated to enhance the abundance of *Clostridium*, due to its capacity to facilitate the fixation of nitrogen and stimulate growth [65]. Consistent with our findings, other studies have corroborated the presence of *Bacillus*, *Pseudomonas*, *Staphylococcus*, and *Clostridium* in lettuce samples [66] or *Pseudomonas* and *Enterococcus* in chives [67]. Although these genera include pathogenic species, they are predominantly composed of environmental saprophytic microorganisms; thus, their detection does not necessarily indicate a higher food safety risk.

On the other hand, a substantial fraction of the dominant microbiome detected at the retail level, particularly psychotropic and opportunistic genera such as *Pseudomonas, Stenotrophomonas, Acinetobacter* and *Enterobacteriaceae*, is not usually associated with soil or plant microbiomes. Therefore, its presence likely reflects post-harvest selection and environmental acquisition rather than the original field-associated community [14].

### 3.2. Eukaryotic Microbiome

Although prior studies have focused primarily on bacterial communities or field-associated fungal assemblages, data concerning the eukaryotic microbiome of fresh vegetables at the retail level remains limited. The present study aims to address this knowledge gap by characterizing the eukaryotic microbial communities associated with organically and conventionally produced vegetables at the point of consumer exposure.

A total of 15,435,129 raw reads were obtained following sequencing. After the cleaning process, a total of 15,427,745 reads were retained for further analysis, and after the rarefaction process, a total of 4,443,600 reads were obtained, resulting in a total of 29,624 ASVs per sample (Table S3). It should be noted that certain samples could not be included in the analysis due to the low number of reads (Table S1).

**Figure 3:**
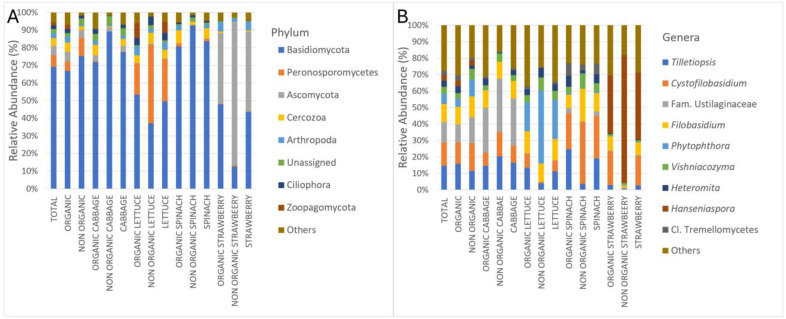
Relative abundance (%) of eukaryotic communities in organic and non-organic products at the phylum (**A**) and genus (**B**) levels.

At the phylum level (Figure 3A), the most abundant taxa were Basidiomycota (69.20%) and Peronosporomycetes (6.54%). A comparable study carried out by Sequino et al. (2022) [68] in 47 samples in the ‘green leafy vegetables’ group identified Basidiomycota as one of the most prevalent phyla. The authors also reported the presence of other phyla, including Ascomycota, Chytridiomycota, Mucoromycota and Zoopagomycota, which were also detected in the present study in low abundances. At the genus level, *Tilletiopsis* (14.64%) was the most prevalent, followed by *Cystofilobasidium* (14.16%) (Figure 3B).

The Kruskal–Wallis analysis revealed that there were no statistically significant differences in alpha diversity, neither in the number of observed features between organic and non-organic products (Figure S3), nor in the results of the Faith’s physiological diversity index (Kruskal–Wallis test, *p* = 0.116) (Table S3). The comparison of sample types within the same matrix demonstrated only a significant discrepancy between organic and non-organic cabbage samples (Kruskal–Wallis test, *p* = 0.0338; Figure S4, Table S4), with organic cabbage samples exhibiting a higher number of observed features (Figure S4).

Analysis of beta diversity revealed distinct patterns in microbial community structure across samples. Pairwise comparisons (Tables S6–S9) indicated no significant difference in the relationship between organic and non-organic samples, neither at the phylum (Table S6) nor genus (Table S8) levels. Furthermore, microbial community composition exhibited high similarity regardless of the matrix type, suggesting that differences did not substantially influence taxonomic distribution.

**Figure 4:**
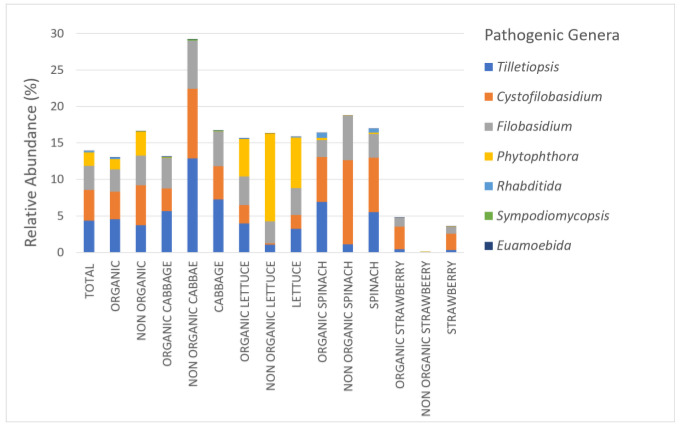
Relative abundance (%) of clinically relevant eukaryotic genera in organic and non-organic products.

The present study identified potentially pathogenic eukaryotic genera of both plant and human relevance (Figure 4). The predominant genus *Tilletiopsis*, which has recently undergone reclassification under the name *Gjaerumia* [69], was detected at a relative frequency of 14,64%*. Candida* and *Naegleria*, both of which include clinically significant species, were detected at low abundances of 0.37% and 0.078%, respectively. *Candida* is of particular significance, due to its ability to cause life-threatening systemic diseases in immunocompromised individuals [70,71]. The genus *Naegleria* includes the species *Naegleria fowleri*, responsible for a fatal disease of the central nervous system [72,73]. However, this species was not detected in the present study, as the taxonomic resolution achieved was limited to the genus level.

### 3.3. Bacterial Microbiome Associated with FLA

Despite extensive evidence on the role of free-living amoebae as reservoirs of pathogenic bacteria in environmental and clinical settings, their contribution to foodborne exposure—particularly through the carriage of viable intracellular bacteria—remains poorly explored and FLA are not commonly addressed in food microbiome studies.

In this work, a total of 11,696,545 raw reads were obtained after sequencing. Following the cleaning process, a total of 11,465,046 reads were retained for further analysis. After the rarefaction process, a total of 1,799,008 reads were obtained, resulting in a total of 13,228 ASVs per sample. Due to the low number of reads available for inclusion (Tables S1 and S3), some samples were not included in the analysis.

**Figure 5:**
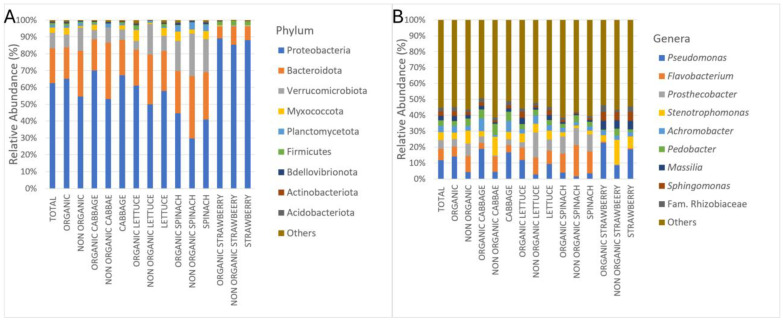
Relative abundance (%) of bacterial communities associated with free-living amoebae isolated from organic and non-organic products at the phylum (**A**) and genus (**B**) levels.

At the phylum level, Proteobacteria (62.53%), Bacteroidota (20.70%), and Verrucomicrobiota (9.19%) were identified (Figure 5A). At the genus level, *Pseudomonas* (11.81%) emerged as the most frequently detected taxon followed by *Flavobacterium* (7.19%) and *Prosthecobacter* (5.57%) (Figure 5B). Similar taxonomic profiles have previously been reported in FLA isolated from fresh organic produce, where *Pseudomonas* and *Flavobacterium* were also identified as the most abundant genera associated with FLA [74]. Alpha diversity analyses revealed no significant differences in the number of observed features between organic and non-organic products (Figure S5). Furthermore, Faith’s phylogenetic diversity index corroborated the absence of statistically significant variation (*p* = 0.529; Table S4). When samples were stratified by matrix type, however, statistically significant differences were observed between organic and non-organic cabbages (*p* = 0.024) and strawberries (*p* = 0.0035) (Figure S6; Table S5).

Regarding beta diversity, pairwise comparisons (Tables S6–S9) demonstrated no significant correlation between organic and non-organic samples, a pattern consistent across both phylum (Table S6) and genus (Table S8) classifications, indicating a largely homogeneous FLA-associated microbiome across production systems and matrix types.

**Figure 6:**
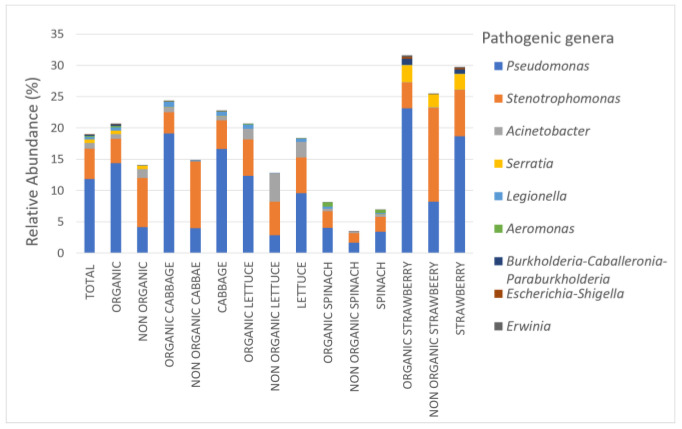
Relative abundance (%) of clinically relevant bacterial genera associated with free-living amoebae isolated from organic and non-organic products.

Several potentially pathogenic bacterial genera were identified within the FLA microbiome, including *Pseudomonas* (11.00%), *Stenotrophomonas* (4.90%), and *Acinetobacter* (0.90%). In addition, the presence of *Legionella* (less than 0.4%) and *Escherichia–Shigella* (less than 0.01%) was detected (Figure 6). The ecological affinity of these genera with FLA [75] suggests a stable association which seems to be independent of cultivation practices. Previous studies have demonstrated that FLA can act as intracellular reservoirs for pathogenic bacteria, protecting them from environmental stressors and disinfection processes [76]. This intracellular persistence may enhance the survival and transmission of pathogens in the food chain, posing a potential risk to public health.

The consistent presence of FLA in all analyzed samples underscores their ubiquity in food production environments and highlights their potential role as overlooked reservoirs of microbial diversity, including opportunistic human pathogens.

### 3.4. Pathogen Viability in FLA Microbiomes

As mentioned above, FLA are unicellular eukaryotic microorganisms that have been observed in a variety of environments, including both aquatic and terrestrial environments. It has been demonstrated that FLA have the capacity to act as a reservoir for several parasitic and pathogenic bacteria, which are resistant and maintain viability inside them, thus highlighting their potential as a reservoir and/or means of dissemination [77,78]. Although metagenomic sequencing provides valuable insights into the taxonomic composition of microbial communities associated with free-living amoebae (FLAs), it does not determine bacterial viability. DNA-based methods can detect both live and dead cells, what can lead to the overestimation of certain taxa. To overcome this issue, DVC-FISH was used to identify viable, pathogenic bacteria specifically within the FLA. This technique combines direct viable counting with fluorescence in situ hybridization to enable the visualization and quantification of metabolically active cells that retain the ability to grow [39]. It has been previously applied for the detection of pathogenic bacteria, including *Escherichia coli* [79] and *Vibrio* spp. [80] in environmental matrices. By targeting specific taxa using tailored probes, DVC-FISH allows a more accurate assessment of the potential health risks associated with viable intracellular pathogens harboured by free-living amoebae (FLA) [81,82].

*L. monocytogenes*, *Helicobacter* spp., *Salmonella* spp. and *Pseudomonas* spp. [83,84,85] were selected for DVC-FISH analysis due to their recognized relevance as foodborne pathogens and their documented association with free-living amoebae (FLA) in environmental and food matrices [85,86,87,88,89].

Within the FLA, no viable *L. monocytogenes*, *Salmonella* spp., nor *Helicobacter* spp. cells were detected in any sample. Otherwise, 95.5% of the samples tested positive for viable *Pseudomonas* spp. (Figure S7). These results are in accordance with the composition of bacterial microbiome previously identified within the amoebae, as neither *L. monocytogenes*, nor *Helicobacter* spp. or *Salmonella* spp. were detected through metagenomic analysis, while *Pseudomonas* was frequently detected.

Several studies have confirmed the presence of *Pseudomonas* in plants [90,91,92]. In addition, a study was conducted to identify *Pseudomonas* in well water samples which contained amoebae [93], demonstrating its ability to maintain viability within them. FLA impose a selective barrier that favours taxa with specific adaptations, such as *Pseudomonas* spp. [94,95]. By integrating high-throughput microbiome profiling with single-cell viability assays, this study reveals how ecological selection mediated by free-living amoebae constrains functional persistence within fresh produce microbiomes, identifying *Pseudomonas* spp. as a selectively favoured intracellular strategist. This emphasizes the need for improved sanitation practices in post-harvest environments and for monitoring bacteria associated with amoebae.

## 4. Conclusions

This study provides a comprehensive analysis of the microbial communities associated with organic and conventionally grown fresh produce, with a particular focus on food safety and the potential public health risks posed by pathogenic microorganisms.

Our results show that the microbiome structure is similar at both the phylum and genus levels between organic and conventional products. This microbiological homogeneity is consistent with previous studies that have shown that the final microbial load of fresh produce depends more on post-harvest processes such as handling, storage, distribution and the sales environment than on the agricultural system itself.

It is noteworthy that the genus *Stenotrophomonas* exhibited a markedly increased prevalence in conventional produce. This finding suggests that organic farming practices may play a role in mitigating the establishment of certain opportunistic pathogens. Conversely, genera such as *Bacillus*, *Enterococcus*, and *Clostridium* exhibited a higher abundance in organic samples, a phenomenon that can be attributed to the use of biopesticides and animal manure.

Analysis of the microbiome associated with FLA revealed no significant differences in diversity between organic and non-organic samples. However, the detection of metabolically active *Pseudomonas* spp. in 94.5% of samples via DVC-FISH underscores the potential for FLAs to harbour and protect pathogens from environmental stressors and disinfection processes.

Overall, our results suggest that, although organic and conventional products have a similar final microbiome and equivalent biological risk, organic products could be considered preferable in terms of reducing chemical risk. Meanwhile, strategies for controlling microbiological risk should focus on improving post-harvest practices throughout the food chain.

## Data Availability

The original contributions presented in this study are included in the article/Supplementary Materials. Further inquiries can be directed to the corresponding authors.

## Supplementary Materials

The following supporting information can be downloaded at: https://www.mdpi.com/article/10.3390/foods15122230/s1, Table S1: Study samples; Table S2; Probes used in DVC-FISH; Table S3: Sequencing output and ASV recovery for the three microbiome datasets; Table S4: Comparison of alpha diversity between organic and non organic fresh produce samples using Faith’s Phylogenetic Diversity index; Table S5: Comparison of alpha diversity between organic and non organic fresh produce samples by type of matrix using Faith’s Phylogenetic Diversity index; Table S6:Analysis of similarities (ANOSIM) comparing microbial community composition between organic and non organic samples at the phylum level; Table S7: Analysis of similarities (ANOSIM) comparing microbial community composition between organic and non organic samples by type of matrix at the phylum level; Table S8: Analysis of similarities (ANOSIM) comparing microbial community composition between organic and non organic samples at the genus level; Table S9: Analysis of similarities (ANOSIM) comparing microbial community composition between organic and non organic samples by type of matrix at the genus level; Table S10: SIMPER analysis of selected genus-level comparisons showing meaningful ANOSIM separation between organic and non organic samples. Only genus-level comparisons showing statistically significant ANOSIM results together with moderate or higher R values were included. Genera contributing cumulatively up to approximately 70% of the average Bray-Curtis dissimilarity are shown. Mean transformed abundance refers to the average abundance values after square-root transformation. Av.Diss: average contribution of each genus to the overall Bray-Curtis dissimilarity; Diss/SD: ratio between average dissimilarity and its standard deviation; Contribution (%): percentage contribution of each genus to the average dissimilarity; Cumulative contribution (%): cumulative percentage contribution. Dataset*B: Bacterial microbiome; F: FLA-associated bacterial microbiome; Figure S1: Alpha diversity analysis of the bacterial microbiome between organic and non organic products. (A) Rarefaction curves. (B) Faith’s Phylogenetic Diversity index; Figure S2: Alpha diversity analysis of the bacterial microbiome between different types of organic and non organic products. (A) Rarefaction curves. (B) Faith’s Phylogenetic Diversity index; Figure S3: Alpha diversity analysis of the eukaryotic microbiome between organic and non organic samples. (A) Rarefaction curves. (B) Faith’s Phylogenetic Diversity index; Figure S4: Alpha diversity of the eukaryotic microbiome between different types of organic and non organic samples. (A) Rarefaction curves. (B) Faith’s Phylogenetic Diversity index; Figure S5: Alpha diversity analysis of the FLA bacterial microbiome between organic and non organic samples. (A) Rarefaction curves. (B) Faith’s Phylogenetic Diversity index; Figure S6: Alpha diversity analysis of the FLA bacterial microbiome between different types of organic and non organic products. (A) Rarefaction curves. (B) Faith’s Phylogenetic Diversity index; Figure S7: Viable *Pseudomonas* spp. by DVC-FISH at 40x magnification. Detection of the green universal EUB probe in sample EF129 (A) and EF145 (C). Detection of the *Pseudomonas* spp. specific red probe in sample EF129 (B) and EF145 (D).

## References

[1] Vega-Zamora M., Parras-Rosa M., Torres-Ruiz F.J. (2020). You are what you eat: The relationship between values and organic food consumption. Sustainability.

[2] Kyrylov Y., Thompson S.R., Hranovska V., Krykunova V. (2018). The world trends of organic production and consumption. Manag. Theory Stud. Rural Bus. Infrastruct. Dev..

[3] Gamage A., Gangahagedara R., Gamage J., Jayasinghe N., Kodikara N., Suraweera P., Merah O. (2023). Role of organic farming for achieving sustainability in agriculture. Farming Syst..

[4] Gundala R.R., Singh A. (2021). What motivates consumers to buy organic foods? Results of an empirical study in the United States. PLoS ONE.

[5] Wu Y., Wu B., Ma Y., Wang M., Feng Q., He Z. (2023). Rapid Discrimination of Organic and Non-Organic Leafy Vegetables (Water Spinach, Amaranth, Lettuce, and Pakchoi) Using VIS-NIR Spectroscopy, Selective Wavelengths, and Linear Discriminant Analysis. Appl. Sci..

[6] Jiang B., Pang J., Li J., Mi L., Ru D., Feng J., Li X., Zhao A., Cai L. (2024). The effects of organic food on human health: A systematic review and meta-analysis of population-based studies. Nutr. Rev..

[7] Nikonova N., Ronzhin A., Kostyaev A. (2023). Global Trends in the Production and Consumption of Organic Products. Agriculture Digitalization and Organic Production.

[8] Abadias M., Usall J., Anguera M., Solsona C., Viñas I. (2008). Microbiological quality of fresh, minimally-processed fruit and vegetables, and sprouts from retail establishments. Int. J. Food Microbiol..

[9] Jasper J., Elmore J.S., Wagstaff C. (2021). Determining the quality of leafy salads: Past, present and future. Postharvest Biol. Technol..

[10] Lorente-Mento J.M., Valverde J.M., Serrano M., Pretel M.T. (2022). Fresh-cut salads: Consumer acceptance and quality parameter evolution during storage in domestic refrigerators. Sustainability.

[11] Grand View Research (2024). Europe Packaged Salad Market Size, Share & Trends Analysis Report by Product (Vegetarian, Non-Vegetarian), by Processing (Organic, Conventional), by Type, by Distribution Channel, by Country, and Segment Forecasts, 2024–2030.

[12] Pilelienė L., Tamulienė V. (2021). Consumer attitudes and behavior towards organic products: Evidence from the Lithuanian market. J. Entrep. Manag. Innov..

[13] Rahman S.M.E., Mele M.A., Lee Y.T., Islam M.Z. (2021). Consumer Preference, Quality, and Safety of Organic and Conventional Fresh Fruits, Vegetables, and Cereals. Foods.

[14] Srisamran J., Atwill E.R., Chuanchuen R., Jeamsripong S. (2022). Detection and analysis of indicator and pathogenic bacteria in conventional and organic fruits and vegetables sold in retail markets. Food Qual. Saf..

[15] Barril P.A., Oteiza J.M., Pardo J., Leotta G.A., Signorini M.L. (2022). Meta-analysis of the prevalence of the main human pathogens in vegetables, with emphasis on lettuce. Food Res. Int..

[16] Kwon H., Lim D.J., Choi C. (2025). Prevention of foodborne viruses and pathogens in fresh produce and root vegetables. Adv. Food Nutr. Res..

[17] Taban B.M., Halkman A.K. (2011). Do leafy green vegetables and their ready-to-eat [RTE] salads carry a risk of foodborne pathogens?. Anaerobe.

[18] Soler L., Moreno-Mesonero L., Jimenez-Belenguer A., Castillo M.Á., Zornoza A., García-Ferrús M., García-Hernandez J., Moreno Y. (2025). Characterization of microbial communities and antibiotic resistance in the water–soil–vegetable interface of a small-scale organic field. Sci. Hortic..

[19] Black Z., Balta I., Black L., Naughton P.J., Dooley J.S.G., Corcionivoschi N. (2021). The fate of foodborne pathogens in manure treated soil. Front. Microbiol..

[20] Nurdin G.M., Patandjengi B., Kuswinanti T. (2025). Comparative Metagenomic Analysis of Rice Phyllosphere Bacterial Communities Under Semi-Organic and Non-Organic Farming Systems. Biocatal. Agric. Biotechnol..

[21] Acharya M., Ashworth A.J., Yang Y., Burke J.M., Lee J.A., Acharya R.S. (2021). Soil microbial diversity in organic and non-organic pasture systems. PeerJ.

[22] Tatsika S., Karamanoli K., Karayanni H., Genitsaris S. (2019). Metagenomic characterization of bacterial communities on ready-to-eat vegetables and effects of household washing on their diversity and composition. Pathogens.

[23] Alegbeleye O., Boas D.M.V., Sant’Ana A.S. (2025). Harnessing the microbiota of vegetables and ready-to-eat (RTE) vegetables for quality and safety. Food Res. Int..

[24] Greub G., Raoult D. (2004). Microorganisms resistant to free-living amoebae. Clin. Microbiol. Rev..

[25] Thomas V., McDonnell G., Denyer S.P., Maillard J.Y. (2010). Free-living amoebae and their intracellular pathogenic microorganisms: Risks for water quality. FEMS Microbiol. Rev..

[26] Fatemi M., Niyyati M., Karamati S.A., Mirjalali H. (2024). Isolation and Molecular Identification of Vahlkampfiidae and *Vermamoeba vermiformis* from Fresh Vegetables: A Neglected Source of Infections. Iran. J. Parasitol..

[27] Moreno-Mesonero L., Soler L., Amorós I., Moreno Y., Ferrús M.A., Alonso J.L. (2023). Protozoan parasites and free-living amoebae contamination in organic leafy green vegetables and strawberries from Spain. Food Waterborne Parasitol..

[28] Cook N., Paton C.A., Wilkinson N., Nichols R.A.B., Barker K., Smith H.V. (2006). Towards standard methods for the detection of *Cryptosporidium parvum* on lettuce and raspberries. Part 2: Validation. Int. J. Food Microbiol..

[29] Moreno-Mesonero L., Hortelano I., Moreno Y., Ferrús M.A. (2020). Evidence of viable *Helicobacter pylori* and other bacteria of public health interest associated with free-living amoebae in lettuce samples by next generation sequencing and other molecular techniques. Int. J. Food Microbiol..

[30] Moreno Y., Moreno-Mesonero L., Amorós I., Pérez R., Morillo J.A., Alonso J.L. (2018). Multiple identification of most important waterborne protozoa in surface water used for irrigation purposes by 18S rRNA amplicon-based metagenomics. Int. J. Hyg. Environ. Health.

[31] Bolyen E., Rideout J.R., Dillon M.R., Bokulich N.A., Abnet C.C., Al-Ghalith G.A., Alexander H., Alm E.J., Arumugam M., Asnicar F. (2019). Reproducible, interactive, scalable and extensible microbiome data science using QIIME 2. Nat. Biotechnol..

[32] Callahan B.J., McMurdie P.J., Rosen M.J., Han A.W., Johnson A.J.A., Holmes S.P. (2016). DADA2: High-resolution sample inference from Illumina amplicon data. Nat. Methods.

[33] Quast C., Pruesse E., Yilmaz P., Gerken J., Schweer T., Yarza P., Perplies J., Glöckner F.O. (2013). The SILVA ribosomal RNA gene database project: Improved data processing and web-based tools. Nucleic Acids Res..

[34] Ríos-Castro R., Cabo A., Teira E., Cameselle C., Gouveia S., Payo P., Novoa B., Figueras A. (2023). High-throughput sequencing as a tool for monitoring prokaryote communities in a wastewater treatment plant. Sci. Total Environ..

[35] Bray J.R., Curtis J.T. (1957). An ordination of the upland forest communities of southern Wisconsin. Ecol. Monogr..

[36] Clarke K.R., Gorley R.N., Somerfield P.J., Warwick R.M. (2014). Change in Marine Communities: An Approach to Statistical Analysis and Interpretation.

[37] Anderson M.J., Gorley R.N., Clarke K.R. (2008). PRIMER + for PERMANOVA: Guide to Software and Statistical Methods.

[38] Clarke K.R., Gorley R.N. (2015). PRIMER v7: User Manual/Tutorial.

[39] Besnard V., Federighi M., Cappelier J.M. (2000). Development of a direct viable count procedure for the investigation of VBNC state in *Listeria monocytogenes*: DVC procedure in *Listeria monocytogenes*. Lett. Appl. Microbiol..

[40] Piqueres P., Moreno Y., Alonso J.L., Ferrús M.A. (2006). A combination of direct viable count and fluorescent in situ hybridization for estimating *Helicobacter pylori* cell viability. Res. Microbiol..

[41] Rengifo-Herrera J.A., Castaño O.L., Sanabria I.J. (2013). Culturability and viability of *Salmonella typhimurium* during photo-Fenton process at pH 5.5 under solar simulated irradiation. J. Water Resour. Prot..

[42] Amann R.I., Krumholz L., Stahl D.A. (1990). Fluorescent-oligonucleotide probing of whole cells for determinative, phylogenetic, and environmental studies in microbiology. J. Bacteriol..

[43] Santiago P., Jiménez-Belenguer A., García-Hernández J., Estellés R.M., Hernández M., Castillo M.A., Ferrús M.A., Moreno Y. (2018). High prevalence of *Salmonella* spp. in wastewater reused for irrigation assessed by molecular methods. Int. J. Hyg. Environ. Health.

[44] Amann R., Ludwig W., Schulze R., Spring S., Moore E., Schleifer K.-H. (1996). rRNA-Targeted Oligonucleotide Probes for the Identification of Genuine and Former Pseudomonads. Syst. Appl. Microbiol..

[45] Chan V., Crocetti G., Grehan M., Zhang L., Danon S., Lee A., Mitchell H. (2005). Visualization of *Helicobacter* Species Within the Murine Cecal Mucosa Using Specific Fluorescence In Situ Hybridization. Helicobacter.

[46] Moreno Y., Ballesteros L., García-Hernández J., Santiago P., González A., Ferrús M.A. (2011). Specific detection of viable *Listeria monocytogenes* in Spanish wastewater treatment plants by Fluorescent In Situ Hybridization and PCR. Water Res..

[47] Moreno Y., Ferrús M.A., Medina E., Jiménez A., Martínez M., Hernández J. (2001). Direct detection of *Helicobacter pylori* in clinical samples by in situ hybridization. Clin. Microbiol. Infect..

[48] Tandon A., Jabeen F., Talwar S., Sakashita M., Dhir A. (2021). Facilitators and inhibitors of organic food buying behavior. Food Qual. Prefer..

[49] Leff J.W., Fierer N. (2013). Bacterial communities associated with the surfaces of fresh fruits and vegetables. PLoS ONE.

[50] Environmental Working Group (2025). EWG’s 2025 Shopper’s Guide to Pesticides in Produce™: Dirty Dozen™.

[51] Artimová R., Játiová M., Baumgartnerová J., Lipková N., Petrová J., Maková J., Javoreková S., Hleba L., Medová J., Medo J. (2023). Microbial communities on samples of commercially available fresh-consumed leafy vegetables and small berries. Horticulturae.

[52] Franco-Frías E., Mercado-Guajardo V., Merino-Mascorro A., Pérez-Garza J., Heredia N., León J.S., Jaykus L.A., Dávil-Aviña J., García S. (2021). Analysis of bacterial communities by 16S rRNA gene sequencing in a melon-producing agro-environment. Microb. Ecol..

[53] Oliveira M., Usall J., Viñas I., Anguera M., Gatius F., Abadias M. (2010). Microbiological quality of fresh lettuce from organic and conventional production. Food Microbiol..

[54] Willis A. (2019). Rarefaction Alpha Diversity, and Statistics. Front. Microbiol..

[55] Walters K.E., Martiny J.B.H. (2020). Alpha-, beta-, and gamma-diversity of bacteria varies across habitats. PLoS ONE.

[56] Lee S., Kim S., Sunwoo J.S. (2023). Otogenic Enterobacter cloacae meningitis complicated with pneumocephalus. Encephalitis.

[57] Bagińska N., Cieślik M., Górski A., Jończyk-Matysiak E. (2021). El papel de *A. baumannii* resistente a antibióticos en la patogénesis de la infección del tracto urinario y el potencial de su tratamiento con el uso de terapia con bacteriófagos. Antibióticos.

[58] Li Y., Roberts J.A., Walker M.M., Aslan A.T., Harris P.N., Sime F.B. (2024). The global epidemiology of ventilator-associated pneumonia caused by multi-drug resistant *Pseudomonas aeruginosa: A* systematic review and meta-analysis. Int. J. Infect. Dis..

[59] Wang N., Tang C., Wang L. (2022). Risk factors for acquired *Stenotrophomonas maltophilia* pneumonia in intensive care unit: A systematic review and meta-analysis. Front. Med..

[60] Aiyedun S.O., Onarinde B.A., Swainson M., Dixon R.A. (2021). Foodborne outbreaks of microbial infection from fresh produce in Europe and North America: A systematic review of data from this millennium. Int. J. Food Sci. Technol..

[61] Kumar A., Rithesh L., Kumar V., Raghuvanshi N., Chaudhary K., Abhineet, Pandey A.K. (2023). *Stenotrophomonas* in diversified cropping systems: Friend or foe?. Front. Microbiol..

[62] Li D., Wong C.H., Seet M.F., Kuan N. (2019). Isolation, characterization, and inactivation of *Stenotrophomonas maltophilia* from leafy green vegetables and urban agriculture systems. Front. Microbiol..

[63] Khan A.R., Mustafa A., Hyder S., Valipour M., Rizvi Z.F., Gondal A.S., Yousuf Z., Iqbal R., Daraz U. (2022). *Bacillus* spp. as bioagents: Uses and application for sustainable agriculture. Biology.

[64] Adetunji C.O., Anani O.A., Olaniyan O.T., Inobeme A., Olisaka F.N., Uwadiae E.O., Obayagbona O.N., Soni R., Suyal D.C., Bhargava P., Goel R. (2021). Recent trends in organic farming. Microbiological Activity for Soil and Plant Health Management.

[65] Lim S.C., Knight D.R., Moono P., Foster N.F., Riley T.V. (2020). *Clostridium difficile* in soil conditioners, mulches and garden mixes with evidence of a clonal relationship with historical food and clinical isolates. Environ. Microbiol. Rep..

[66] Lee W., Kim M.H., Park J., Kim Y.J., Kim E., Heo E.J., Kim S.H., Kim G., Shin H., Kim S.H. (2023). Seasonal Changes in the Microbial Communities on Lettuce (*Lactuca sativa* L.) in Chungcheong-do, South Korea. J. Microbiol. Biotechnol..

[67] Seo D.W., Yum S.J., Lee H.R., Kim S.M., Jeong H.G. (2021). Microbiota analysis and microbiological hazard assessment in chinese chive (*Allium tuberosum* Rottler) depending on retail types. J. Microbiol. Biotechnol..

[68] Sequino G., Valentino V., Torrieri E., De Filippis F. (2022). Specific microbial communities are selected in minimally-processed fruit and vegetables according to the type of product. Foods.

[69] Pernice M.C., Forn I., Logares R., Massana R. (2024). A fungi hotspot deep in the ocean: Explaining the presence of Gjaerumia minor in equatorial Pacific bathypelagic waters. Sci. Rep..

[70] Li H., Miao M.X., Jia C.L., Cao Y.B., Yan T.H., Jiang Y.Y., Yang F. (2022). Interactions between *Candida albicans* and the resident microbiota. Front. Microbiol..

[71] Lopes J.P., Lionakis M.S. (2022). Pathogenesis and virulence of *Candida albicans*. Virulence.

[72] Phung N.T.N., Pham H.T., Tran T.T., Dinh V.H., Tran N.M., Tran N.A.N., Ngo M.Q.N., Nguyen H.T.T.N., Tran D.K., Le T.K.T. (2025). *Naegleria fowleri*: Portrait of a Cerebral Killer. Diagnostics.

[73] Alanazi A., Younas S., Ejaz H., Alruwaili M., Alruwaili Y., Mazhari B.B.Z., Atif M., Junaid K. (2025). Advancing the understanding of *Naegleria fowleri*: Global epidemiology, phylogenetic analysis, and strategies to combat a deadly pathogen. J. Infect. Public Health.

[74] Soler L., Moreno Y., Moreno-Mesonero L., Amorós I., Alonso J.L., Ferrús M.A. (2023). Microbiome of free-living amoebae (FLA) isolated from fresh organic produce: Potential risk to consumers?. Foods.

[75] Price C.T.D., Hanford H.E., Al-Quadan T., Santic M., Shin C.J., Da’as M.S.J., Abu Kwaik Y. (2024). Amoebae as training grounds for microbial pathogens. mBio.

[76] Shi Y., Queller D.C., Tian Y., Zhang S., Yan Q., He Z., He Z., Wu C., Wang C., Shu L. (2021). The ecology and evolution of amoeba-bacterium interactions. Appl. Environ. Microbiol..

[77] Bornier F., Zas E., Potheret D., Laaberki M.H., Coupat-Goutaland B., Charpentier X. (2021). Environmental free-living amoebae can predate on diverse antibiotic-resistant human pathogens. Appl. Environ. Microbiol..

[78] Sousa-Ramos D., Reyes-Batlle M., Bellini N.K., Rodríguez-Expósito R.L., Martín-Real C., Piñero J.E., Lorenzo-Morales J. (2022). Pathogenic free-living amoebae from water sources in Cape Verde. Parasitol. Res..

[79] Garcia-Armisen T., Servais P. (2004). Combining direct viable count (DVC) and fluorescent in situ hybridisation (FISH) to enumerate viable *E. coli* in rivers and wastewaters. Water Sci. Technol..

[80] Girard L., Peuchet S., Servais P., Henry A., Charni-Ben-Tabassi N., Baudart J. (2017). Spatiotemporal Dynamics of Total Viable *Vibrio* spp. in a NW Mediterranean Coastal Area. Microbes Environ..

[81] Moreno-Mesonero L., Moreno Y., Alonso J.L., Ferrús M.A. (2016). DVC-FISH and PMA-qPCR techniques to assess the survival of *Helicobacter pylori* inside *Acanthamoeba castellanii*. Res. Microbiol..

[82] Moreno Y., Moreno-Mesonero L., García-Hernández J. (2019). DVC-FISH to identify potentially pathogenic *Legionella* inside free-living amoebae from water sources. Environ. Res..

[83] Kanarek P., Bogiel T., Breza-Boruta B. (2022). Risk of legionellosis: A general overview of habitats of *Legionella* spp. in Europe. Environ. Sci. Pollut. Res..

[84] Liébana-Rodríguez M., Recacha-Villamor E., Díaz-Molina C., Pérez-Palacios P., Martín-Hita L., Enríquez-Maroto F., Gutiérrez-Fernández J. (2024). Outbreaks by *Klebsiella oxytoca* in neonatal intensive care units: Analysis of an outbreak in a tertiary hospital and systematic review. Enfermedades Infecc. Microbiol. Clin. (Engl. Ed.).

[85] Gartley S., Anderson-Coughlin B., Sharma M., Kniel K.E. (2022). *Listeria monocytogenes* in irrigation water: An assessment of outbreaks, sources, prevalence, and persistence. Microorganisms.

[86] Osek J., Lachtara B., Wieczorek K. (2022). *Listeria monocytogenes*–how this pathogen survives in food-production environments?. Front. Microbiol..

[87] Moreno-Mesonero L., Moreno Y., Alonso J.L., Ferrús M.A. (2017). Detection of viable *Helicobacter pylori* inside free-living amoebae in wastewater and drinking water samples from Eastern Spain. Environ. Microbiol..

[88] Popa G.L., Papa M.I. (2021). *Salmonella* spp. infection-a continuous threat worldwide. Germs.

[89] Mooney R., Richardson K., Rodgers K., Giammarini E., Williams R., Kelly S., Amaeze N., Inkster T., Henriquez F.L., Mackay W. (2024). *Acanthamoebae* as a Protective Reservoir for *Pseudomonas aeruginosa* in a Clinical Environment. J. Hosp. Infect..

[90] Fanelli F., Caputo L., Quintieri L. (2021). Phenotypic and genomic characterization of *Pseudomonas putida* ITEM 17297 spoiler of fresh vegetables: Focus on biofilm and antibiotic resistance interaction. Curr. Res. Food Sci..

[91] Ruiz-Roldán L., Rojo-Bezares B., Lozano C., López M., Chichón G., Torres C., Sáenz Y. (2021). Occurrence of *Pseudomonas* spp. in raw vegetables: Molecular and phenotypical analysis of their antimicrobial resistance and virulence-related traits. Int. J. Mol. Sci..

[92] Fakhkhari P., Tajeddin E., Azimirad M., Salmanzadeh-Ahrabi S., Abdi-Ali A., Nikmanesh B., Eshrati B., Mehdi-Gouya M., Owlia P., Reza-Zali M. (2022). Involvement of *Pseudomonas aeruginosa* in the occurrence of community and hospital acquired diarrhea, and its virulence diversity among the stool and the environmental samples. Int. J. Environ. Health Res..

[93] Potgieter N., Van der Loo C., Barnard T.G. (2021). Co-existence of free-living amoebae and potential human pathogenic bacteria isolated from rural household water storage containers. Biology.

[94] Leong W., Poh W., Williams J., Lutz C., Hoque M.M., Poh Y.H., Yee B.Y.K., Chua C., Givskov M., Sanderson-Smith M. (2022). Adaptation to an Amoeba Host Leads to *Pseudomonas aeruginosa* Isolates with Attenuated Virulence. Appl. Environ. Microbiol..

[95] Matz C., Kjelleberg S. (2005). Off the hook—How bacteria survive protozoan grazing. Trends Microbiol..

