# Supplementary material for: Retail-Level Microbiomes of Organic and Conventional Fresh Produce: A Multi-Kingdom Analysis of Amoeba-Associated Bacterial Viability
Source: Foods. 2026 Jun 20;15(12):2230. doi: 10.3390/foods15122230 (PMC13297928; doi:10.3390/foods15122230)
Supplement: Supplementary file 1 [file foods-15-02230-s001.zip › foods-4340692-supplementary.pdf]

Retail-Level Microbiomes of Organic and Conventional Fresh Produce: A Multi Kingdom Analysis of Amoeba Associated Bacterial Viability

Soler, L.[a,] Moreno-Mesonero, L.[a,b,*], García-Hernández, J.[b], García-Ferrús, M.[b], Zornoza, A., Moreno, Y.[a, *]

[a] Instituto de Ingeniería del Agua y Medio Ambiente (IIAMA), Universitat Politècnica de València, Camino de Vera s/n, 46022 València, Spain

[b] Centro Avanzado de Microbiología Aplicada, Universitat Politècnica de València, Camino de Vera s/n, 46022 València, Spain

[c]H2OCITIES, SL. Arte Mayor de la Seda, 15, 46950 Xirivella, Valencia, Spain

[1] These authors share first authorship

*: Corresponding author (ymoren@upv.es)

Table S1. Study samples

Sample number	Bacterial microbiome sample ID	Eukaryotic microbiome sample ID	Bacterial microbiome associated with FLA sample ID	TYPE (ORGANIC/ NON ORGANIC)
**1**	C1b	C1e	C1f	ORGANIC
**2**	C2b	C2e	C2f	ORGANIC
**3**	C3b	C3e	C3f	ORGANIC
**4**	C4b	C4e	C4f	ORGANIC
**5**	C5b	C5e	C5f	ORGANIC
**6**	C6b	C6e	C6f	ORGANIC
**7**	C7b	C7e	C7f	ORGANIC
**8**	C8b	C8e	C8f	ORGANIC
**9**	C9b	C9e	C9f	ORGANIC
**10**	C10b	C10e	C10f	ORGANIC
**11**	C11b	C11e	C11f	ORGANIC
**12**	C12b	C12e	C12f	ORGANIC
**13**	C13b	C13e	C13f	ORGANIC
**14**	C14b	C14e	C14f	ORGANIC
**15**	C15b	C15e	C15f	ORGANIC
**16**	C16b	C16e	C16f	ORGANIC
**17**	C17b	C17e	C17f	ORGANIC
**18**	NA*	C18e	C18f	ORGANIC
**19**	C19b	C19e	C19f	ORGANIC
**20**	C20b	C20e	C20f	ORGANIC
**21**	C21b	C21e	C21f	ORGANIC
**22**	C22b	C22e	C22f	ORGANIC
**23**	C23b	C23e	C23f	ORGANIC
**24**	C24b	C24e	C24f	ORGANIC

Sample number	Bacterial microbiome sample ID	Eukaryotic microbiome sample ID	Bacterial microbiome associated with FLA sample ID	TYPE (ORGANIC/ NON ORGANIC)
**25**	C25b	C25e	C25f	ORGANIC
**26**	C26b	C26e	C26f	ORGANIC
**27**	C27b	C27e	C27f	ORGANIC
**28**	C28b	C28e	C28f	ORGANIC
**29**	C29b	C29e	NA	ORGANIC
**30**	C30b	C30e	C30f	ORGANIC
**31**	C31b	C31e	C31f	NON ORGANIC
**32**	C32b	C32e	C32f	ORGANIC
**33**	C33b	C33e	C33f	NON ORGANIC
**34**	NA	C34e	C34f	NON ORGANIC
**35**	C35b	C35e	C35f	NON ORGANIC
**36**	C36b	C36e	C36f	NON ORGANIC
**37**	C37b	C37e	C37f	NON ORGANIC
**38**	C38b	C38e	NA	NON ORGANIC
**39**	C39b	C39e	NA	NON ORGANIC
**40**	C40b	C40e	NA	NON ORGANIC
**41**	C41b	C41e	NA	NON ORGANIC
**42**	Sp1b	Sp1e	Sp1f	ORGANIC
**43**	Sp2b	Sp2e	Sp2f	ORGANIC
**44**	Sp3b	Sp3e	NA	ORGANIC
**45**	Sp4b	Sp4e	Sp4f	ORGANIC
**46**	Sp5b	Sp5e	Sp5f	ORGANIC
**47**	Sp6b	Sp6e	Sp6f	ORGANIC
**48**	Sp7b	Sp7e	Sp7f	ORGANIC
**49**	Sp8b	Sp8e	Sp8f	ORGANIC
**50**	Sp9b	Sp9e	NA	NON ORGANIC
**51**	Sp10b	Sp10e	Sp10f	ORGANIC
**52**	Sp11b	Sp11e	Sp11f	ORGANIC
**53**	Sp12b	Sp12e	Sp12f	ORGANIC
**54**	Sp13b	Sp13e	NA	ORGANIC
**55**	Sp14b	Sp14e	NA	ORGANIC
**56**	Sp15b	Sp15e	Sp15f	ORGANIC
**57**	Sp16b	Sp16e	Sp16f	ORGANIC
**58**	Sp17b	Sp17e	Sp17f	ORGANIC
**59**	Sp18b	Sp18e	Sp18f	ORGANIC
**60**	Sp19b	Sp19e	Sp19f	ORGANIC
**61**	Sp20b	Sp20e	Sp20f	ORGANIC
**62**	Sp21b	Sp21e	Sp21f	ORGANIC

Sample number	Bacterial microbiome sample ID	Eukaryotic microbiome sample ID	Bacterial microbiome associated with FLA sample ID	TYPE (ORGANIC/ NON ORGANIC)
**63**	Sp22b	Sp22e	Sp22f	ORGANIC
**64**	Sp23b	Sp23e	Sp23f	ORGANIC
**65**	Sp24b	Sp24e	Sp24f	ORGANIC
**66**	Sp25b	Sp25e	Sp25f	ORGANIC
**67**	NA	Sp26e	Sp26f	ORGANIC
**68**	NA	Sp27e	Sp27f	ORGANIC
**69**	NA	Sp28e	Sp28f	ORGANIC
**70**	Sp29b	Sp29e	Sp29f	ORGANIC
**71**	Sp30b	Sp30e	Sp30f	ORGANIC
**72**	Sp31b	Sp31e	Sp31f	ORGANIC
**73**	Sp32b	Sp32e	Sp32f	NON ORGANIC
**74**	Sp33b	Sp33e	Sp33f	NON ORGANIC
**75**	Sp34b	Sp34e	Sp34f	ORGANIC
**76**	Sp35b	Sp35e	Sp35f	NON ORGANIC
**77**	Sp36b	Sp36e	Sp36f	NON ORGANIC
**78**	Sp37b	Sp37e	Sp37f	NON ORGANIC
**79**	Sp38b	Sp38e	Sp38f	NON ORGANIC
**80**	Sp39b	Sp39e	Sp39f	NON ORGANIC
**81**	Sp40b	Sp40e	Sp40f	NON ORGANIC
**82**	Sp41b	Sp41e	Sp41f	NON ORGANIC
**83**	St1b	St1e	St1f	ORGANIC
**84**	St2b	St2e	St2f	ORGANIC
**85**	St3b	St3e	NA	ORGANIC
**86**	St4b	St4e	St4f	ORGANIC
**87**	St5b	St5e	St5f	ORGANIC
**88**	St6b	St6e	St6f	ORGANIC
**89**	St7b	St7e	St7f	ORGANIC
**90**	St8b	St8e	St8f	ORGANIC
**91**	St9b	St9e	St9f	NON ORGANIC
**92**	St10b	St10e	St10f	NON ORGANIC
**93**	St11b	St11e	St11f	NON ORGANIC
**94**	St12b	St12e	St12f	ORGANIC
**95**	St13b	St13e	St13f	ORGANIC
**96**	St14b	St14e	St14f	ORGANIC
**97**	St15b	St15e	St15f	ORGANIC
**98**	St16b	St16e	St16f	ORGANIC
**99**	St17b	St17e	St17f	ORGANIC
**100**	St18b	St18e	St18f	ORGANIC

Sample number	Bacterial microbiome sample ID	Eukaryotic microbiome sample ID	Bacterial microbiome associated with FLA sample ID	TYPE (ORGANIC/ NON ORGANIC)
**101**	St19b	St19e	St19f	ORGANIC
**102**	St20b	St20e	St20f	ORGANIC
**103**	St21b	St21e	St21f	ORGANIC
**104**	St22b	St22e	St22f	ORGANIC
**105**	St23b	St23e	St23f	ORGANIC
**106**	St24b	St24e	St24f	ORGANIC
**107**	St25b	St25e	St25f	ORGANIC
**108**	St26b	St26e	St26f	NON ORGANIC
**109**	St27b	St27e	St27f	NON ORGANIC
**110**	St28b	St28e	St28f	NON ORGANIC
**111**	St29b	St29e	St29f	NON ORGANIC
**112**	St30b	St30e	St30f	NON ORGANIC
**113**	St31b	NA	St31f	NON ORGANIC
**114**	L1b	L1e	L1f	ORGANIC
**115**	L2b	L2e	L2f	ORGANIC
**116**	L3b	L3e	L3f	ORGANIC
**117**	L4b	L4e	NA	ORGANIC
**118**	L5b	L5e	L5f	ORGANIC
**119**	L6b	L6e	L6f	ORGANIC
**120**	L7b	L7e	L7f	ORGANIC
**121**	L8b	L8e	NA	ORGANIC
**122**	L9b	L9e	NA	ORGANIC
**123**	L10b	L10e	L10f	ORGANIC
**124**	L11b	L11e	NA	NON ORGANIC
**125**	L12b	L12e	L12f	ORGANIC
**126**	L13b	L13e	L13f	ORGANIC
**127**	L14b	L14e	L14f	ORGANIC
**128**	L15b	L15e	L15f	ORGANIC
**129**	L16b	L16e	NA	ORGANIC
**130**	L17b	L17e	NA	ORGANIC
**131**	L18b	L18e	L18f	ORGANIC
**132**	L19b	NA	L19f	ORGANIC
**133**	L20b	L20e	L20f	ORGANIC
**134**	L21b	L21e	L21f	ORGANIC
**135**	L22b	L22e	NA	ORGANIC
**136**	L23b	L23e	L23f	ORGANIC
**137**	L24b	L24e	L24f	ORGANIC
**138**	L25b	L25e	L25f	ORGANIC

Sample number	Bacterial microbiome sample ID	Eukaryotic microbiome sample ID	Bacterial microbiome associated with FLA sample ID	TYPE (ORGANIC/ NON ORGANIC)
**139**	L26b	L26e	L26f	ORGANIC
**140**	L27b	L27e	L27f	NON ORGANIC
**141**	L28b	L28e	L28f	ORGANIC
**142**	L29b	L29e	L29f	ORGANIC
**143**	L30b	L30e	L30f	ORGANIC
**144**	L31b	L31e	L31f	ORGANIC
**145**	L32b	L32e	L32f	NON ORGANIC
**146**	L33b	L33e	L33f	NON ORGANIC
**147**	L34b	L34e	L34f	NON ORGANIC
**148**	L35b	L35e	L35f	NON ORGANIC
**149**	L36b	L36e	L36f	ORGANIC
**150**	L37b	L37e	L37f	NON ORGANIC
**151**	L38b	L38e	L38f	NON ORGANIC
**152**	L39b	L39e	L39f	NON ORGANIC
**153**	L40b	L40e	L40f	NON ORGANIC
**154**	L41b	L41e	L41f	NON ORGANIC

C: Cabbage; Sp: Spinach; St: Strawberry; L: Lettuce

b: bacteria analysis; e: eukaryotic analysis; f: bacterial microbiome associated with free-living amoebae analysis

NA*: Not available

Table S2. Probes used in DVC-FISH

Probe	Sequence (5'-3')	Reference	Dye
**EUB338 Universal Bacterial Probe (EUB338-I, EUB338-II, EUB338-III)**	EUB338-I: GCT GCC TCC CGT AGG AGT<br>EUB338-II: GCA GCC ACC CGT AGG TGT<br>EUB338-III: GCT GCC ACC CGT AGG TGT	[42]	FLUOS (Green)
***Salmonella* spp. (SA23)**	CAC TTC ACC TAC GTG TCA	[43]	CY3 (Red)
***Pseudomonas* spp. (RYD208)**	AAT CGC GCG AGG CCT TAC	[44]	CY3 (Red)
***Helicobacter* spp. (HEL717)**	AGG TCG CCT TCG CAA TGA GTA	[45]	CY3 (Red)
***Listeria monocytogenes* (Lmon)**	CTA TCC ATT GTA GCA CGT G	[46]	CY3 (Red)

Table S3. Sequencing output and ASV recovery for the three microbiome datasets. Total raw reads, reads remaining after quality filtering, reads retained after rarefaction, and the number of amplicon sequence variants (ASVs) obtained for the bacterial microbiome, eukaryotic microbiome, and free-living amoeba (FLA)-associated bacterial microbiome datasets.

	Bacterial microbiome	Eukaryotic microbiome	Bacterial microbiome associated with FLA
**Raw reads**	14,025,326	15,435,189	11,696,545
**Reads after filtering**	4,595,968	15,427,745	11,465,046
**Total reads after rarefaction**	181,184	4,443,600	1,799,008
**ASVs after rarefaction**	1,216	29,624	13,228

Table S4. Comparison of alpha diversity between organic and non organic fresh produce samples using Faith's Phylogenetic Diversity index. P-values were obtained using the Kruskal-Wallis test for the bacterial microbiome, eukaryotic microbiome, and FLA-associated bacterial microbiome datasets.

		Bacterial microbiome	Eukaryotic microbiome	Bacterial microbiome associated with FLA
		**p-value**	**p-value**	**p-value**
**NON ORGANIC**	**ORGANIC**	0.007	0.116	0.529

Table S5. **Comparison of alpha diversity between organic and non organic fresh produce samples by type of matrix using Faith's Phylogenetic Diversity index.** P-values were obtained using the Kruskal-Wallis test for the bacterial microbiome, eukaryotic microbiome, and FLA-associated bacterial microbiome datasets.

		Bacterial microbiome	Eukaryotic microbiome	Bacterial microbiome associated with FLA
		**p-value**	**p-value**	**p-value**
**NON ORGANIC CABBAGE**	**ORGANIC CABBAGE**	0.133	0.0338	0.024
**NON ORGANIC LETTUCE**	**ORGANIC LETTUCE**	0.00064	0.0763	0.064
**NON ORGANIC SPINACH**	**ORGANIC SPINACH**	0.10	0.504	0.065
**NON ORGANIC STRAWBERRY**	**ORGANIC STRAWBERRY**	0.82	0.1594	0.0035

Table S6. Analysis of similarities (ANOSIM) comparing microbial community composition between organic and non organic samples at the phylum level. The number of samples in each group (n), ANOSIM R statistic, and associated p-values are shown for the bacterial microbiome, eukaryotic microbiome, and FLA-associated bacterial microbiome datasets.

	Bacterial microbiome				Eukaryotic microbiome				Bacterial microbiome associated with FLA			
Comparison/Matrix	n organic	n non organic	R	p-value	n organic	n non organic	R	p-value	n organic	n non organic	R	p-value
Non organic vs organic	110	39	0.148	0.002	113	37	0.125	0.003	102	34	0.089	0.027

Table S7. Analysis of similarities (ANOSIM) comparing microbial community composition between organic and non organic samples by type of matrix at the phylum level. The number of samples in each group (n), ANOSIM R statistic, and associated p-values are shown for the bacterial microbiome, eukaryotic microbiome, and FLA-associated bacterial microbiome datasets.

	Bacterial microbiome				Eukaryotic microbiome				Bacterial microbiome associated with FLA			
Comparison/Matrix	n organic	n non organic	R	p-value	n organic	n non organic	R	p-value	n organic	n non organic	R	p-value
Cabbage: non organic vs organic	30	9	0.351	0.001	31	9	0.218	0.017	30	6	0.234	0.055
Lettuce: non organic vs organic	30	11	0.142	0.032	29	10	0.048	0.287	24	10	0.071	0.203
Spinach: non organic vs organic	28	10	0.197	0.022	31	10	0.171	0.0039	27	9	0.096	0.156
Strawberry: non organic vs organic	22	9	0.177	0.024	22	8	0.097	0.172	21	9	-0.028	0.577

Table S8. Analysis of similarities (ANOSIM) comparing microbial community composition between organic and non organic samples at the genus level. The number of samples in each group (n), ANOSIM R statistic, and associated p-values are shown for the bacterial microbiome, eukaryotic microbiome, and FLA-associated bacterial microbiome datasets.

	Bacterial microbiome				Eukaryotic microbiome				Bacterial microbiome associated with FLA			
**Comparison/Matrix**	**n organic**	**n non organic**	**R**	**p-value**	**n organic**	**n non organic**	**R**	**p-value**	**n organic**	**n non organic**	**R**	**p-value**
**Non organic vs organic**	110	39	0.288	0.001	113	37	0.148	0.002	102	34	0.202	0.001

Table S9. Analysis of similarities (ANOSIM) comparing microbial community composition between organic and non organic samples by type of matrix at the genus level. The number of samples in each group (n), ANOSIM R statistic, and associated p-values are shown for the bacterial microbiome, eukaryotic microbiome, and FLA-associated bacterial microbiome datasets.

	Bacterial microbiome				Eukaryotic microbiome				Bacterial microbiome associated with FLA			
**Comparison/Matrix**	**n organic**	**n non organic**	**R**	**p-value**	**n organic**	**n non organic**	**R**	**p-value**	**n organic**	**n non organic**	**R**	**p-value**
**Cabbage: non organic vs organic**	30	9	0.551	0.001	31	9	0.274	0.004	30	6	0.416	0.003
**Lettuce: non organic vs organic**	30	11	0.364	0.001	29	10	0.090	0.130	24	10	0.202	0.0019
**Spinach: non organic vs organic**	28	10	0.226	0.001	31	10	0.295	0.002	27	9	0.421	0.001
**Strawberry: non organic vs organic**	22	9	0.57	0.001	22	8	0.133	0.115	21	9	0.112	0.11

Table S10. SIMPER analysis of selected genus-level comparisons showing meaningful ANOSIM separation between organic and non organic samples. Only genus-level comparisons showing statistically significant ANOSIM results together with moderate or higher R values were included. Genera contributing cumulatively up to approximately 70% of the average Bray-Curtis dissimilarity are shown. Mean transformed abundance refers to the average abundance values after square-root transformation. Av.Diss: average contribution of each genus to the overall Bray-Curtis dissimilarity; Diss/SD: ratio between average dissimilarity and its standard deviation; Contribution (%): percentage contribution of each genus to the average dissimilarity; Cumulative contribution (%): cumulative percentage contribution.

Dataset*B: Bacterial microbiome; F: FLA-associated bacterial microbiome

Dataset* (B/F)	Matrix	Average dissimilarity (%)	Genus	Organic mean transformed abundance	Non organic mean transformed abundance	Av.Diss	Diss/SD	Contribution (%)	Cumulative contribution (%)
B	Cabbage	64.11	*Pseudomonas*	4.18	5.94	7.30	1.29	11.38	11.38
B	Cabbage	64.11	Unassigned genus	3.37	1.65	6.27	1.27	9.79	21.17
B	Cabbage	64.11	*Stenotrophomonas*	1.15	2.42	5.56	1.36	8.66	29.83
B	Cabbage	64.11	*Enterobacter*	1.43	1.18	3.82	1.12	5.96	35.79
B	Cabbage	64.11	*Mesorhizobium*	0.07	1.72	3.74	1.27	5.84	41.63
B	Cabbage	64.11	*Bacillus*	1.63	0.09	3.64	0.87	5.68	47.31
B	Cabbage	64.11	*Bradyrhizobium*	0.31	1.61	3.39	1.43	5.29	52.59
B	Cabbage	64.11	*Variovorax*	0.02	1.40	3.33	0.63	5.19	57.79
B	Cabbage	64.11	*Hydrotalea*	0.00	1.36	3.24	0.67	5.06	62.85
B	Cabbage	64.11	*Serratia*	1.35	0.05	3.04	1.15	4.74	67.58
B	Cabbage	64.11	*Lactococcus*	1.30	0.00	3.02	0.80	4.70	72.29
B	Strawberry	64.20	*Bradyrhizobium*	0.32	3.63	9.21	1.14	14.35	14.35
B	Strawberry	64.20	*Mesorhizobium*	0.04	3.09	8.03	1.13	12.51	26.86
B	Strawberry	64.20	Unassigned genus	3.17	1.95	5.20	1.47	8.09	34.96
B	Strawberry	64.20	*Pantoea*	2.48	1.30	5.07	1.44	7.89	42.85
B	Strawberry	64.20	*Pseudomonas*	3.42	2.33	4.60	1.22	7.16	50.00
B	Strawberry	64.20	*Serratia*	2.04	0.47	4.18	1.18	6.52	56.52
B	Strawberry	64.20	*Lactococcus*	1.38	1.09	3.91	1.35	6.09	62.61
B	Strawberry	64.20	*Enterobacter*	0.62	1.54	3.52	0.95	5.48	68.09

Dataset* (B/F)	Matrix	Average dissimilarity (%)	Genus	Organic mean transformed abundance	Non organic mean transformed abundance	Av.Diss	Diss/SD	Contribution (%)	Cumulative contribution (%)
B	Strawberry	64.20	*Bacillus*	1.65	0.65	3.47	1.43	5.41	73.50
B	Lettuce	45.66	*Stenotrophomonas*	1.25	4.61	8.32	1.76	18.22	18.22
B	Lettuce	45.66	*Acinetobacter*	1.17	2.14	3.99	0.88	8.73	26.95
B	Lettuce	45.66	*Pseudomonas*	5.30	5.05	3.96	1.09	8.68	35.63
B	Lettuce	45.66	*Pantoea*	2.29	2.55	3.34	1.29	7.31	42.95
B	Lettuce	45.66	Unassigned genus 1	1.56	2.40	3.28	1.51	7.19	50.14
B	Lettuce	45.66	*Enterobacter*	0.23	1.51	3.20	1.70	7.01	57.15
B	Lettuce	45.66	*Exiguobacterium*	1.19	0.56	3.08	1.04	6.74	63.89
B	Lettuce	45.66	Unassigned genus 2	1.23	0.65	3.03	0.93	6.63	70.52
F	Cabbage	59.47	*Pseudomonas*	3.58	1.75	4.16	0.94	7.00	7.00
F	Cabbage	59.47	*Sphingomonas*	0.89	2.38	3.50	1.03	5.88	12.88
F	Cabbage	59.47	*Stenotrophomonas*	1.63	2.48	3.28	1.18	5.52	18.40
F	Cabbage	59.47	*Flavobacterium*	1.55	2.35	2.94	1.36	4.94	23.34
F	Cabbage	59.47	*Pedobacter*	1.97	1.94	2.74	1.24	4.61	27.95
F	Cabbage	59.47	*Achromobacter*	2.45	0.95	2.69	1.15	4.53	32.47
F	Cabbage	59.47	*Aquabacterium*	1.58	0.44	2.27	1.10	3.81	36.29
F	Cabbage	59.47	*Prosthecobacter*	1.14	0.33	1.93	0.71	3.25	39.53
F	Cabbage	59.47	*Verrucomicrobium*	0.38	0.88	1.88	0.63	3.17	42.70
F	Cabbage	59.47	*Dyadobacter*	1.07	1.26	1.83	1.05	3.08	45.78
F	Cabbage	59.47	*Massilia*	1.20	0.68	1.62	1.21	2.72	48.50
F	Cabbage	59.47	*Brevifollis*	0.14	0.78	1.58	0.50	2.65	51.15
F	Cabbage	59.47	*Allorhizobium-Neorhizobium-Pararhizobium-Rhizobium*	1.47	1.06	1.54	1.33	2.59	53.74
F	Cabbage	59.47	*Emticicia*	0.22	0.94	1.52	0.60	2.55	56.29

Dataset* (B/F)	Matrix	Average dissimilarity (%)	Genus	Organic mean transformed abundance	Non organic mean transformed abundance	Av.Diss	Diss/SD	Contribution (%)	Cumulative contribution (%)
F	Cabbage	59.47	*Alkanindiges*	0.91	0.03	1.48	0.73	2.50	58.79
F	Cabbage	59.47	*Roseomonas*	1.23	1.21	1.47	1.25	2.47	61.26
F	Cabbage	59.47	*Duganella*	0.73	0.58	1.45	0.90	2.44	63.70
F	Cabbage	59.47	*Nubsella*	0.32	0.84	1.34	0.83	2.26	65.96
F	Cabbage	59.47	*Pseudorhodoferax*	0.66	0.93	1.26	1.34	2.12	68.08
F	Cabbage	59.47	*Pantoea*	0.62	0.53	1.21	0.96	2.03	70.11
F	Spinach	52.70	*Prosthecobacter*	2.70	1.79	4.65	1.16	8.82	8.82
F	Spinach	52.70	*Flavobacterium*	2.97	3.51	4.37	1.14	8.29	17.11
F	Spinach	52.70	*Verrucomicrobium*	1.38	1.20	2.81	1.04	5.33	22.44
F	Spinach	52.70	*Rheinheimera*	0.87	1.35	2.35	1.28	4.47	26.91
F	Spinach	52.70	*Cellvibrio*	1.72	0.57	2.24	0.97	4.25	31.16
F	Spinach	52.70	*Nannocystis*	1.43	0.44	2.03	0.89	3.85	35.01
F	Spinach	52.70	*Runella*	0.16	1.17	1.94	0.96	3.68	38.68
F	Spinach	52.70	*Pedobacter*	1.37	1.53	1.86	1.13	3.54	42.22
F	Spinach	52.70	*Roseimicrobium*	0.52	0.95	1.72	0.66	3.26	45.48
F	Spinach	52.70	*Pseudomonas*	1.72	1.08	1.70	1.06	3.22	48.70
F	Spinach	52.70	*Achromobacter*	1.42	1.25	1.58	1.14	3.00	51.70
F	Spinach	52.70	*Stenotrophomonas*	1.32	1.00	1.44	1.08	2.74	54.44
F	Spinach	52.70	*Larkinella*	0.43	0.88	1.37	0.96	2.61	57.04
F	Spinach	52.70	*Sphingobacterium*	0.59	0.48	1.25	0.96	2.38	59.42
F	Spinach	52.70	*Massilia*	1.00	0.64	1.22	1.55	2.32	61.74
F	Spinach	52.70	*Dyadobacter*	1.37	1.75	1.14	0.89	2.17	63.90
F	Spinach	52.70	*Variovorax*	0.99	1.00	1.13	1.47	2.14	66.05
F	Spinach	52.70	*Terrimicrobium*	0.22	0.48	1.11	0.49	2.11	68.15

Dataset* (B/F)	Matrix	Average dissimilarity (%)	Genus	Organic mean transformed abundance	Non organic mean transformed abundance	Av.Diss	Diss/SD	Contribution (%)	Cumulative contribution (%)
F	Spinach	52.70	*Allorhizobium-Neorhizobium-Pararhizobium-Rhizobium*	1.13	0.66	1.11	1.21	2.10	70.25

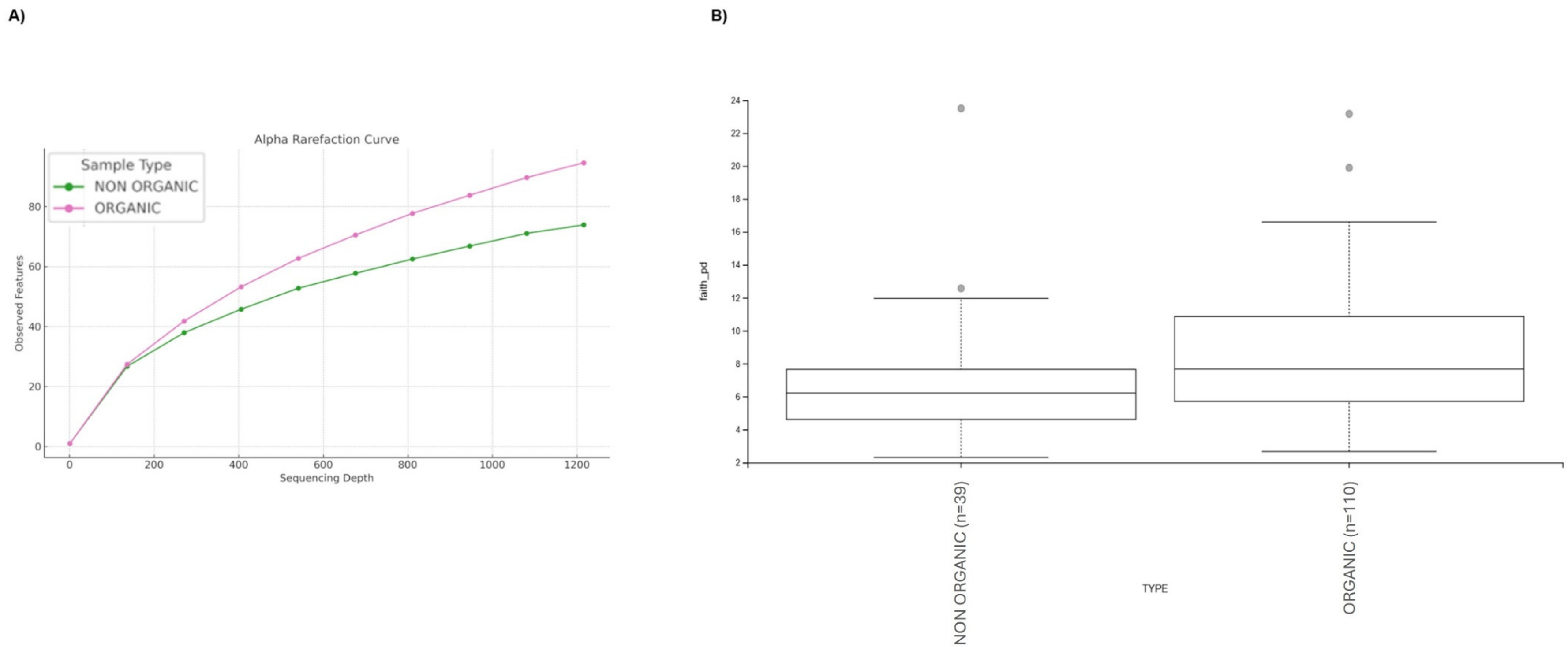

Figure S1. Alpha diversity analysis of the bacterial microbiome between organic and non organic products. **(A)** Rarefaction curves. **(B)** Faith's Phylogenetic Diversity index.

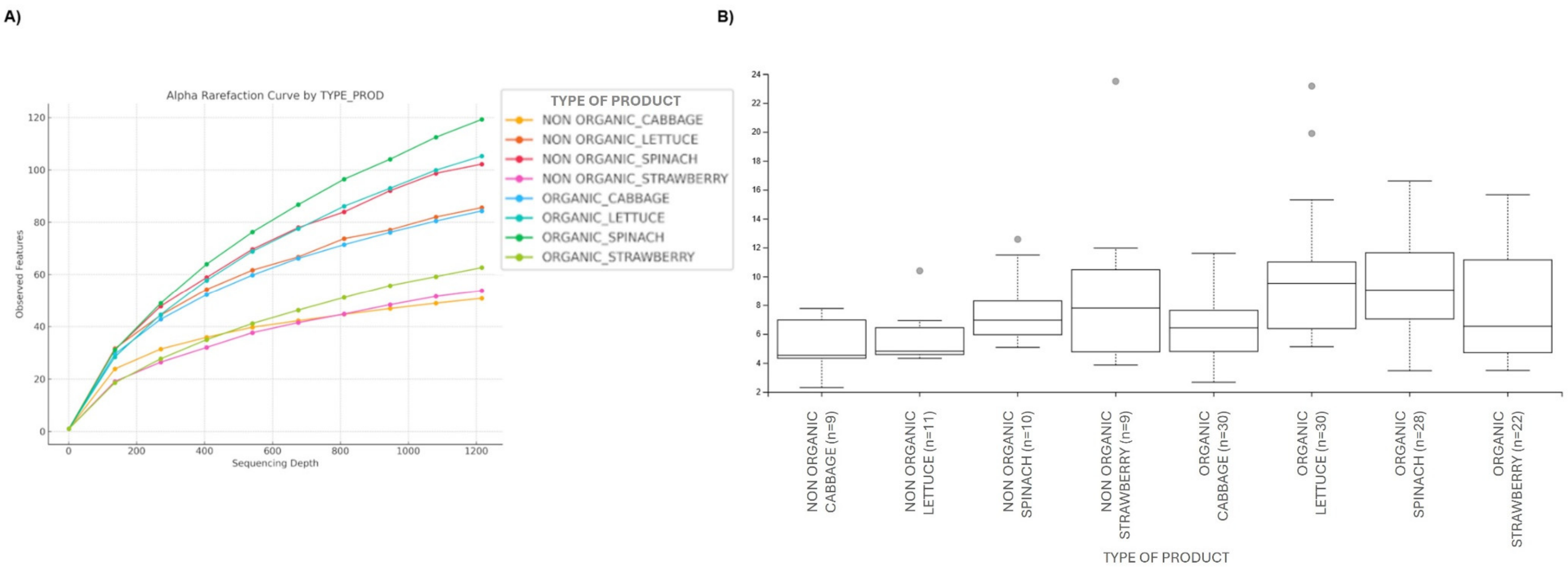

Figure S2. Alpha diversity analysis of the bacterial microbiome between different types of organic and non organic products. **(A)** Rarefaction curves. **(B)** Faith's Phylogenetic Diversity index.

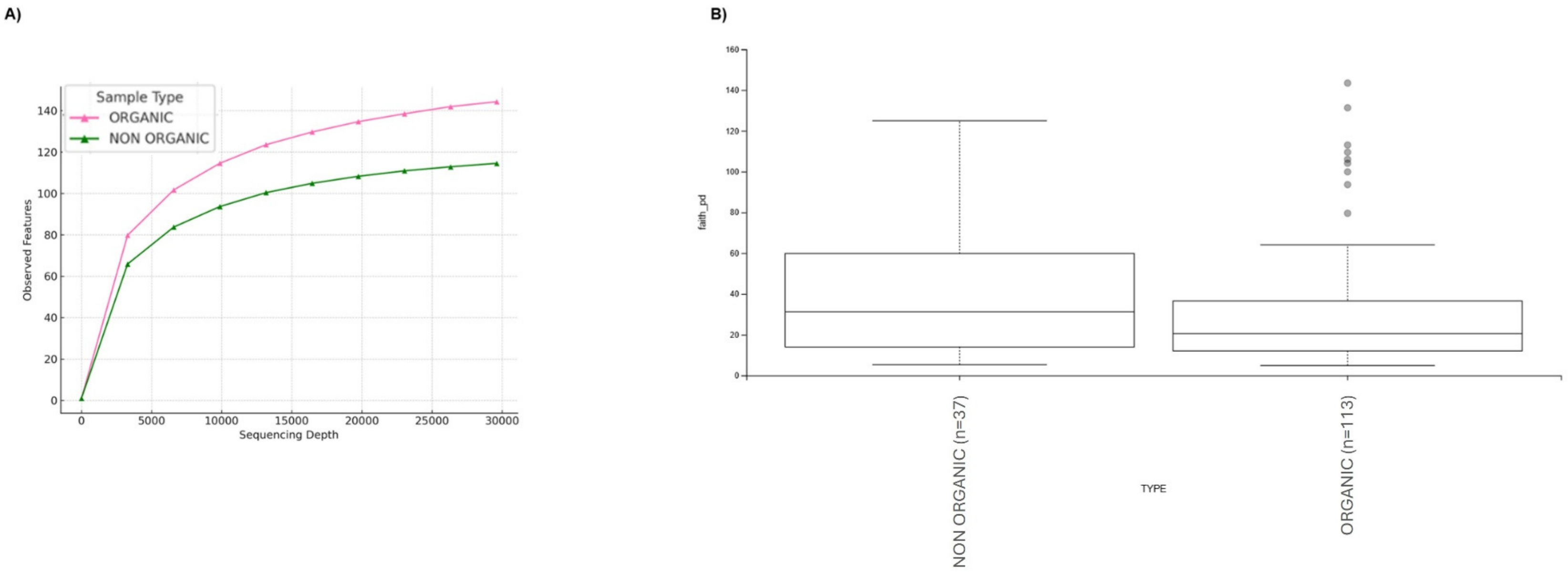

Figure S3. Alpha diversity analysis of the eukaryotic microbiome between organic and non organic samples. **(A)** Rarefaction curves. **(B)** Faith's Phylogenetic Diversity index.

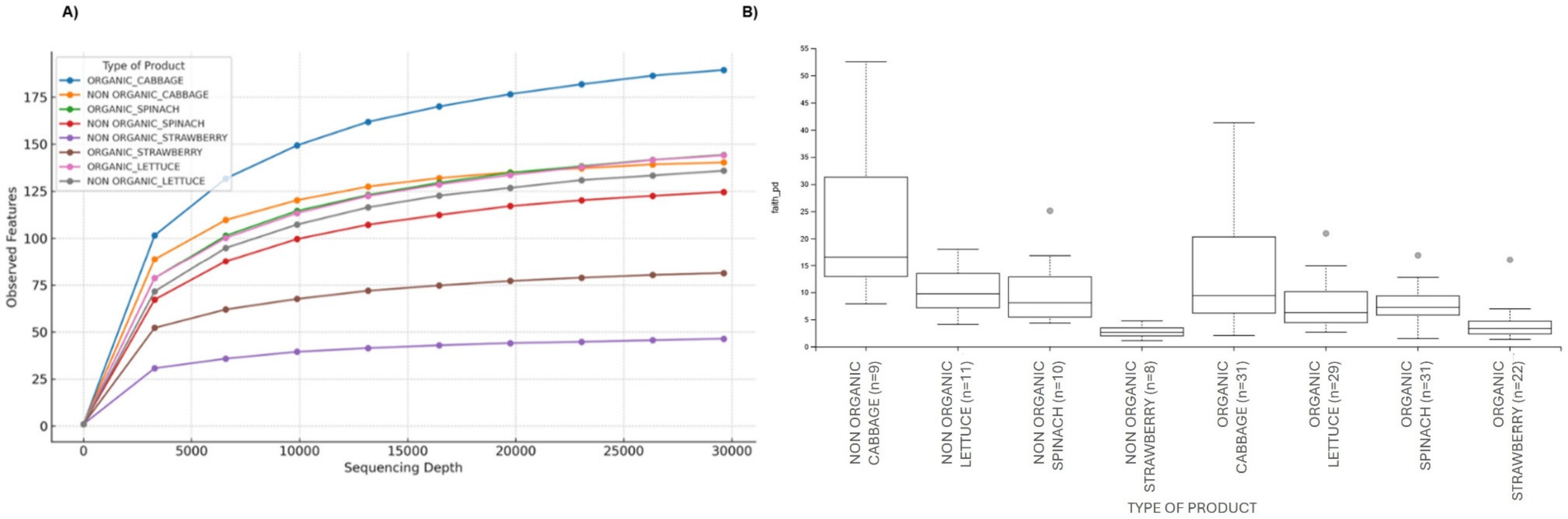

Figure S4. Alpha diversity of the eukaryotic microbiome between different types of organic and non organic samples. **(A)** Rarefaction curves. **(B)** Faith's Phylogenetic Diversity index.

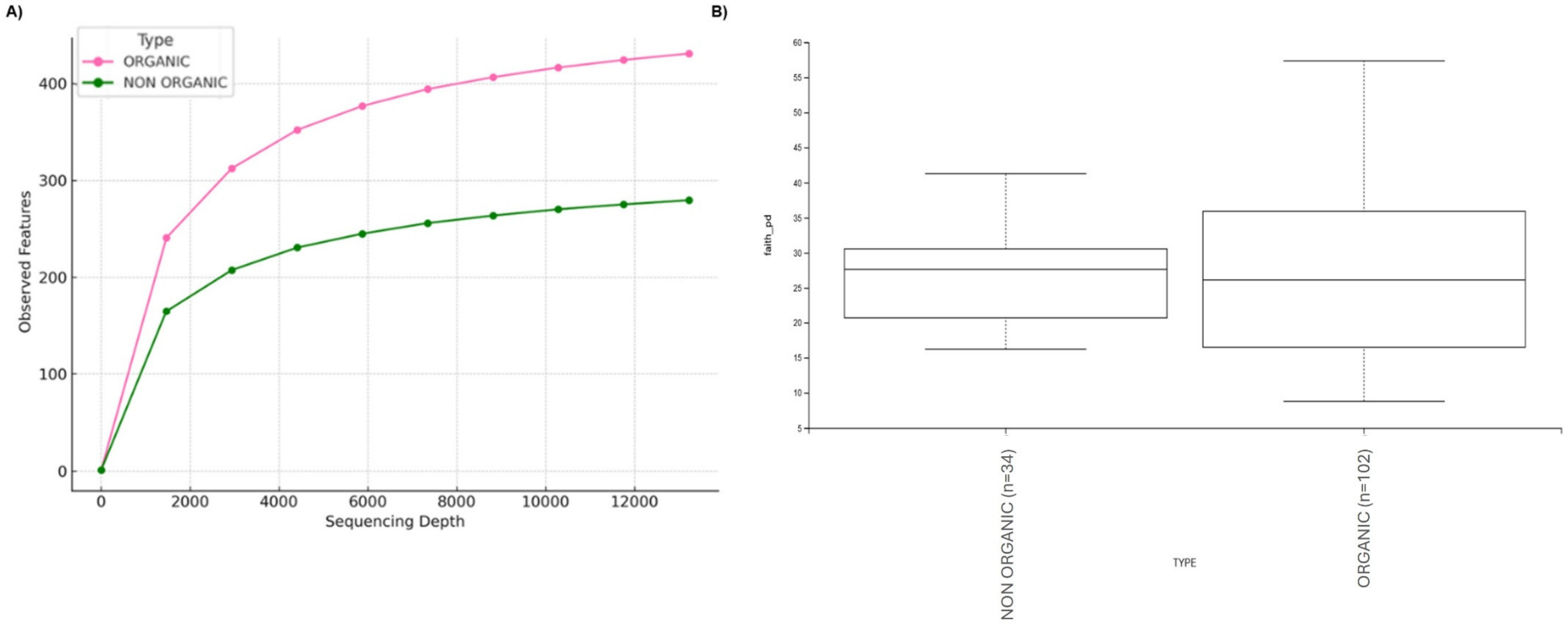

Figure S5. Alpha diversity analysis of the FLA bacterial microbiome between organic and non organic samples. **(A)** Rarefaction curves. **(B)** Faith's Phylogenetic Diversity index.

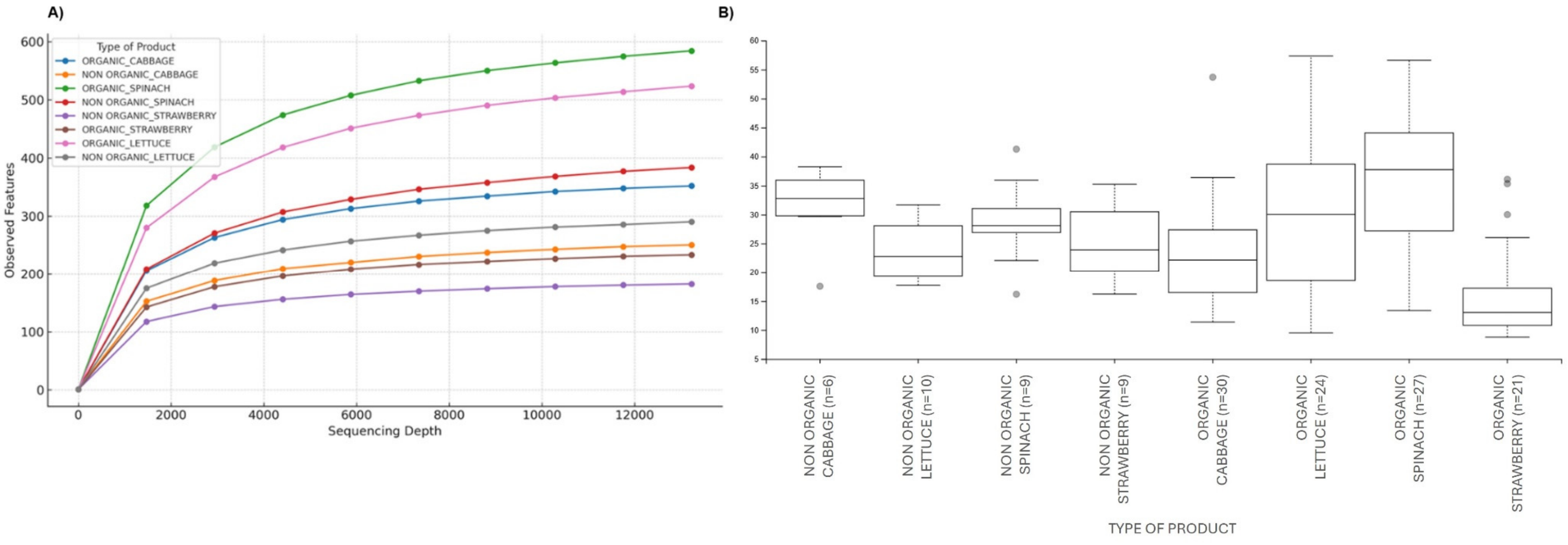

Figure S6. Alpha diversity analysis of the FLA bacterial microbiome between different types of organic and non organic products. **(A)** Rarefaction curves. **(B)** Faith's Phylogenetic Diversity index.

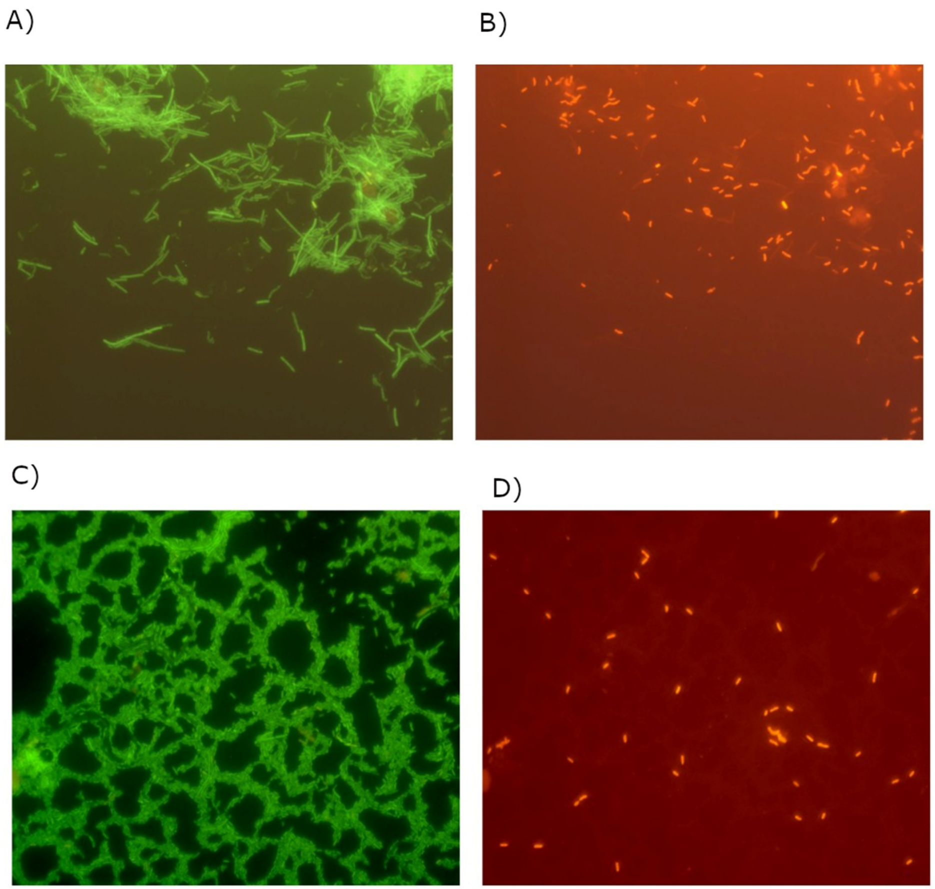

Figure S7. Viable *Pseudomonas* spp. by DVC-FISH. Detection of the green universal EUB probe in sample EF129 **(A)** and EF145 **(C).** Detection of the *Pseudomonas* spp. specific red probe in sample EF129 **(B)** and EF145 **(D).** Images were acquired at ×1000 magnification.
